# Synaptic changes in psychiatric and neurological disorders: state-of-the art of in vivo imaging

**DOI:** 10.1038/s41386-024-01943-x

**Published:** 2024-08-12

**Authors:** Oliver Howes, Julia Marcinkowska, Federico E. Turkheimer, Richard Carr

**Affiliations:** 1 Department of Psychosis Studies, Institute of Psychiatry, Psychology and Neuroscience, King’s College London, London, England; 2 South London & the Maudsley NHS Trust, London, England; 3 London Institute of Medical Sciences, London, England; 4 Department of Neuroimaging, Institute of Psychiatry, Psychology and Neuroscience, King’s College London, London, England

**Keywords:** Neurotransmitters, Diseases of the nervous system, Translational research

## Abstract

Synapses are implicated in many neuropsychiatric illnesses. Here, we provide an overview of in vivo techniques to index synaptic markers in patients. Several positron emission tomography (PET) tracers for synaptic vesicle glycoprotein 2 A (SV2A) show good reliability and selectivity. We review over 50 clinical studies including over 1700 participants, and compare findings in healthy ageing and across disorders, including addiction, schizophrenia, depression, posttraumatic stress disorder, and neurodegenerative disorders, including tauopathies, Huntington’s disease and α-synucleinopathies. These show lower SV2A measures in cortical brain regions across most of these disorders relative to healthy volunteers, with the most well-replicated findings in tauopathies, whilst changes in Huntington’s chorea, Parkinson’s disease, corticobasal degeneration and progressive supranuclear palsy are predominantly subcortical. SV2A PET measures are correlated with functional connectivity across brain networks, and a number of other measures of brain function, including glucose metabolism. However, the majority of studies found no relationship between grey matter volume measured with magnetic resonance imaging and SV2A PET measures. Cognitive dysfunction, in domains including working memory and executive function, show replicated inverse relationships with SV2A measures across diagnoses, and initial findings also suggest transdiagnostic relationships with mood and anxiety symptoms. This suggests that synaptic abnormalities could be a common pathophysiological substrate underlying cognitive and, potentially, affective symptoms. We consider limitations of evidence and future directions; highlighting the need to develop postsynaptic imaging markers and for longitudinal studies to test causal mechanisms.

## Introduction

Most complex brain functions rely on synapses [1]. Unsurprisingly, therefore, synaptic alterations are implicated in many neurological and psychiatric disorders [2, 3]. Synaptic markers have historically been measured postmortem, often utilising immunofluorescence to study the density of synaptic vesicle proteins [4]. However, synaptic changes may occur after death [5], and cause of death may affect synapses [6]. Furthermore, it is not possible to study changes during the course of the disorder or relate them to the development of clinical sequelae in the same individual in postmortem studies. These issues highlight the need for techniques for in vivo investigation of synapses in the brain to enable determination of relationships between synaptic markers and clinical features of illness, and how they change with time. The last decade has seen the development of positron emission tomography (PET) imaging techniques to quantify synaptic proteins, providing the most direct in vivo measures of synaptic markers for use in humans available to date [7]. These PET methods are now being used to study synaptic protein levels in a number of neuropsychiatric disorders, which makes it timely to consider progress to date and future avenues of investigation. In view of this, we first provide an overview of synaptic organisation and the elements indexed by current neuroimaging methods. Following this, we discuss PET, and the tracers that have been developed to target synaptic proteins. We then review the findings to date in neuropsychiatric disorders, comparing the regions and degree of alterations, and reviewing convergent transdiagnostic SV2A findings in cognition and relationships with other neuroimaging measures. These aspects have not been comprehensively covered by previous reviews of SV2A imaging [8–10] to our knowledge. Finally, we consider the limitations of current approaches and next steps.

## Overview of the synapse

**Fig. 1:**
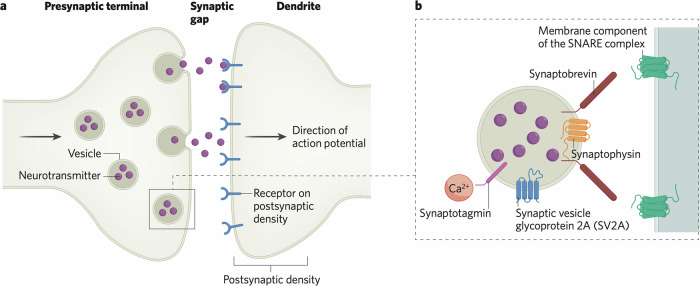
Structure of a typical synapse and synaptic vesicle. **a** Overview of the synapse, showing the presynaptic terminal with vesicles containing neurotransmitter, synaptic gap, and the postsynaptic density with neurotransmitter binding to receptors. Action potentials (arrow) trigger vesicular fusion with the presynaptic membrane to release neurotransmitter (**b**), which then diffuses across the synaptic gap to bind to receptors in the post-synaptic density. **b** Representation of a vesicle primed for fusion with the presynaptic membrane, showing key vesicle proteins. SNARE soluble *N*-ethylamide-sensitive factor attachment protein receptor complex.

The synapse comprises a presynaptic terminal, the synaptic cleft and the postsynaptic density (Fig. 1a). In the presynaptic terminal, calcium influx induced by incoming action potentials causes synaptic vesicles to fuse with the presynaptic terminal membrane and release the neurotransmitter into the synaptic cleft (Fig. 1b). The neurotransmitter diffuses across the cleft and binds to receptors on the postsynaptic density [11]. The binding of neurotransmitter can produce diverse effects, including excitation, inhibition and induction of synaptic plasticity [12]. Consequently, the synapse is the core unit of information processing in the brain [13].

Synaptic vesicles are complex structures, containing protein machinery for packaging vesicles with neurotransmitter [14], responding to calcium influx into the presynaptic terminal to initiate exocytosis, mediated by synaptotagmin, a calcium sensor [15], and for fusing with the synaptic terminal membrane, mediated by the soluble *N*-ethylamide-sensitive factor attachment protein receptor (SNARE) complex of proteins. The SNARE complex comprises a membrane component and the vesicle protein synaptobrevin [16]. Other key proteins mediating these processes include synaptophysin and synaptic vesicle glycoprotein 2 A (SV2A), both of which are thought to control the trafficking of other vesicle proteins such as synaptobrevin and synaptotagmin respectively (Fig. 1B) [8, 17]. Postmortem studies often use immunolabelling of these synaptic proteins [18], particularly synaptophysin, while current PET methods for synaptic imaging use tracers that bind to SV2A. SV2A is, as far as is known, ubiquitously expressed in synapses, making it a good marker of synapses [19]. Visualisation of the distribution of synaptophysin and SV2A shows that the two proteins colocalise in the synapse, at least in mouse brain, suggesting alignment between the primary targets measured with in vitro and in vivo methods respectively [20].

## Synaptic imaging

### Background

Synaptic imaging studies using PET typically estimate one of four outcome measures; volume of distribution (V_T_), distribution volume ratio (DVR), standardised uptake value ratio (SUVR) and nondisplaceable binding potential (BP_ND_). A detailed description of outcome measures is beyond the scope of this review, however a brief summary is present in the Supplementary Materials, and further detail can be found in previous reviews on the subject [7, 21].

The ideal tracer for investigating synaptic density requires a target present in all synapses, but not expressed in other cellular components. While not perfect as a target, synaptic vesicle glycoprotein 2A (SV2A) is a protein that approaches this ideal and for which there are PET tracers available for human imaging [7]. SV2A is a member of the SV2 family of transmembrane vesicle proteins [8], present in presynaptic terminals (Fig. 1) with no evidence for expression in the postsynaptic density. A number of tracers that bind to SV2A with high affinity and selectivity have been developed, summarised in Table 1. One of the first produced was [^11^C]LEV, based on the SV2A modulator levetiracetam [22]. However, [^11^C]LEV has not been used for human studies, due to poor brain uptake [23]. Three other tracers with more rapid brain penetrance have been developed: [^11^C]UCB-A, [^18^F]UCB-H, and [^11^C]UCB-J [24]. The inferior brain uptake of [^11^C]UCB-A relative to the other UCB tracers [25] meant it has not proceeded to human studies [26].

**Table 1:** Summary of characteristics of tracers targeting SV2A.

Tracer	Affinity for SV2A (K_i_, nM)	Measured BP_ND_^a^ (grey matter)	T_max_	First Used in Humans	Test-retest variability (V_T_)
[^11^C]LEV	NI	NI	NI	Not used	NI
[^11^C]UCB-A	1.2 [152]	NI	65 mins [25]	Not used	NI
[^11^C]UCB-J	2.6 [152], 1.5 [153]	1.85–3.7 [35], 2.2–3.7 [30]	10–25 mins [35]	2016 [47]	3–9%; ICC > 0.6 [154]
[^18^F]UCB-H	6.8 [152], 9 [153]	<1 [30]	10 mins [155] (NHP)	2015 [156]	10% [155] (NHP)
[^18^F]SynVesT-1	3.1 [152], 2.2 [153]	2.4–4.3 [35], 2.8–4.6 [30]	5–20 mins [35]	2021 [35]	<9% [34]
[^18^F]SynVest-2	9.6 [152]	1.6–3.0 [30]	7 mins (reported in thalamus and putamen only) [157], 7 mins (dlPFC and putamen only) [30]	2020 [30]	5.5% [157], 4.7–7.2% [29]
[^18^F]SDM-16	0.9 [31] (NHP)	2.8 [31] (NHP)	NI	Not used in humans	7% [31] (NHP)

*CSO* centrum semiovale, *dlPFC* dorsolateral PFC, *ICC* interclass correlation coefficient (given where reported), *NHP* non-human primates, *SV2A* synaptic vesicle glycoprotein 2A, *V*_*T*_ volume of distribution, *BP*_*ND*_ binding potential, *T*_*max*_ time to maximum concentration in the brain, *NI* no information.

^a^Studies use the CSO to estimate *BP*_*ND*_, which may not be a true reference region (see Limitations Section). As such this is not a true value of *BP*_*ND*_.

Of the UCB tracers, [^11^C]UCB-J has higher affinity for SV2A [27] (Table 1) and higher specific signal than [^18^F]UCB-H [27]. Test-retest variability was ≤10% for all the tracers where it has been examined, indicating low within-subject variability (Table 1). These test-retest studies have generally been over 1–7 days. However, one study conducted [^11^C]UCB-J PET scans performed four weeks apart [28] showing comparable low variability to shorter-term studies in three outcome measures, with volume of distribution, distribution volume ratio and nondisplaceable binding potential in total grey matter (GM) showing test-retest values of −7.7 ± 4.3%, −6.6 ± 6.1%, and −8.2 ± 9.6% respectively.

The SynVesT tracers are fluorinated analogues of UCB-J [29]. This allows radiofluorination into [^18^F] tracers, a favourable practical factor for clinical applications, as the slower decay of the tracer allows it to be produced off-site and transported to imaging centres without synthesis facilities. They are also less lipophilic, which may decrease nonspecific binding [30]. However, they are rapidly broken down, with <50% of the parent molecule present at 30 min post-injection [31]. They have since been used in human research [32, 33], with [^18^F]SynVesT-1 showing higher BP_ND_ compared to [^11^C]UCB-J and very rapid kinetics [10, 34, 35]. [^18^F]SynVesT-2 showed lower uptake than [^18^F]SynVesT-1 and [^11^C]UCB-J, with mean BP_ND_ values 42% and 24% lower than these respectively in direct comparison [30], meaning it will probably have lower sensitivity to pick up small changes in SV2A levels than the other tracers.

A seventh tracer, [^18^F]SDM-16, was developed in 2021, based on the molecular structure of [^11^C]UCB-A [31], designed to overcome the metabolic lability of other [^18^F] tracers. It shows the highest affinity for SV2A, measured in rhesus macaques, and comparable BP_ND_ to other tracers (Table 1), but has not yet been used in humans.

### In vivo selectivity of SV2A PET Tracers for SV2a

Blocking studies have been used to evaluate the specificity of PET tracers to SV2A. These involve PET scans using the tracer before and after administration of a drug which binds to the SV2A protein, thus competing with the tracer. Tracers which show a larger decrease between scans are considered more selective [36]. However, even with perfect blocking, residual signal will remain due to nonspecific binding of the tracer, for example to lipids or other macromolecular cellular components [37]. Most such studies use levetiracetam, which has a moderate affinity for human SV2A (K_i_: 6.1–8 μM) [38, 39]. While it shows negligible binding at the SV2 isoforms SV2B and SV2C [22], it has also been shown to bind to N-type voltage gated calcium channels [40] and AMPA receptors [41], although its affinity at these sites has not been reported.

Results from blocking studies are presented in Table 2. Whilst direct comparisons are complicated by studies being performed in different species, [^11^C]UCB-J shows higher displacement at 30 mg/kg levetiracetam than [^18^F]UCB-H at 100 mg/kg, and is roughly on-par with [^18^F]SynVesT-1 at 200 mg/kg dose. [^18^F]SynVesT-1 may slightly outperform [^11^C]UCB-J at a 20 mg/kg dose in humans, showing higher displacement. One study has been performed with [^18^F]SynVest-2, showing comparable reductions in V_T_ to [^18^F]SynVesT-1 following 20 mg/kg levetiracetam [29]. No levetiracetam blocking data were found for [^11^C]UCB-A or [^11^C]LEV. Brivaracetam, another SV2A modulator shows higher affinity for the protein (K_i_ = 225 nM) than levetiracetam [42] and strong evidence of selectivity, with no specific binding observed in SV2A knockout mice [42]. It has been used in one blocking study [25] showing a ~75% decrease in whole-brain [^11^C]UCB-A SUV following brivaracetam administration in mice.

**Table 2:** Results from blocking studies following administration of the SV2A modulator levetiracetam.

Tracer	Change in V_T_ following administration of different doses levetiracetam (% reduction, excluding centrum semiovale)					
	10 mg/kg (59% SV2A occupancy [26])	20 mg/kg (82.5–85.3% SV2A occupancy [35])	30 mg/kg (90% SV2A occupancy [26])	50 mg/kg (97.4% SV2A occupancy [158])	100 mg/kg (SV2A occupancy unknown)	200 mg/kg (100% SV2A occupancy [158]
[^11^C]LEV	NI	NI	NI	NI	NI	NI
[^11^C]UCB-A	NI	NI	NI	NI	NI	NI
[^11^C]UCB-J	41.0–60.4 [26] (NHP)	55.2–67.7 [35] (humans)	58.3–78.3 [26] (NHP)	74.7–78.7 [158] (mice)	NI	84.1–86.7 [158] (mice)
[^18^F]UCB-H	46.2 [159], 25.7–36.8 [43] (rats)	NI	NI	NI	34.4–55.8 [43] (rats)	NI
[^18^F]SynVesT-1	NI	64.7–76.2 [35] (humans)	NI	60.6–78.6 [160] (mice)	NI	73.2–89.5 [160] (mice)
[^18^F]SynVest-2	NI	65.0–70.6% (humans) [29]	NI	NI	NI	NI
[^18^F]SDM-16	NI	NI	57% [31] (NHP)	NI	NI	NI

Data presented show the reduction in V_T_ in the post-dose PET scan compared to the baseline scan. In tracers which have higher selectivity for the SV2A protein, a greater proportion of that tracer will be in competition with levetiracetam at the protein, and therefore these will show greater V_T_ reductions in the post-dose scan. Data from different doses of levetiracetam are presented to allow comparison. Higher doses of levetiracetam are expected to occupy a greater proportion of SV2A protein, thus leading to a greater difference between baseline and post-dose V_T_.

*NHP* non-human primates, *NI* no information, *SV2A* synaptic vesicle glycoprotein 2A.

In vitro studies have also been conducted with [^11^C]UCB-J, finding a 10-fold greater selectivity for human SV2A over SV2C and a 100-fold greater selectivity over SV2B [27], however such studies have not been performed with other tracers. In vitro screening of both [^11^C]UCB-J and [^18^F]UCB-H revealed no significant activity (<50% inhibition at 10 μM) at a standard panel of receptors, ion channels and enzymes, including >55 potential targets [27, 43].

### Validation Studies with SV2A PET Tracers

**Fig. 2:**
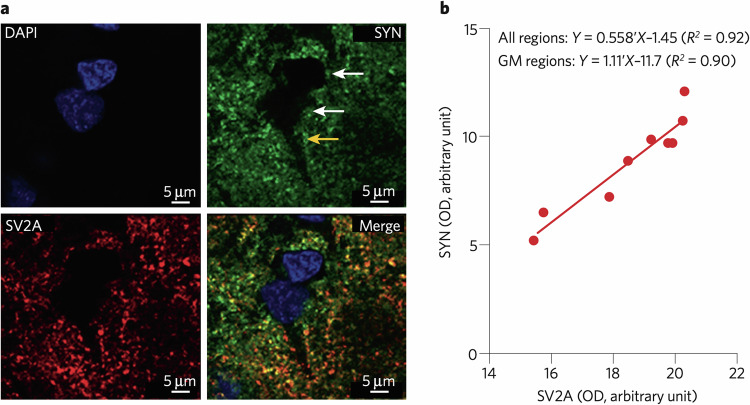
Colocalisation of SV2A and synaptophysin in the baboon brain. **a** High-power confocal microscopy of 4’,6-diamidino-2-phenylindole (DAPI), synaptophysin (SYN), and synaptic vesicle glycoprotein 2A (SV2A) in the grey matter of the baboon brain. Labelling for SYN and SV2A is evident as punctate staining in the neuropil, particularly surrounding neuronal cell bodies and proximal dendrites (yellow arrow), but absent in neuronal cell bodies (white arrows). Nuclei are indicated by the DAPI stain in blue. **b** Correlation between in vitro SV2A and in vitro SYN density in grey matter regions determined using Western blot analyses. Data are nine brain regions. Figure and legend reproduced from Finnema et al. with permission (2016) [47]. GM grey matter, OD optical density.

One key target for validation of these tracers is showing a clear relationship between SV2A tracer binding and established measures of synaptic density. Animal models provide evidence that SV2A tracers can detect synaptic loss, and are sensitive to change. For example, significant reductions in [^11^C]UCB-J [44] and [^18^F]SDM-16 [45] SUVR were detected in the hippocampus of the APP/PS1 mouse model of Alzheimer’s dementia (AD), in which an independent ultramicroscopy study showed significant hippocampal synaptic degeneration [46]. Interestingly, a baboon study investigating both synaptophysin immunolabelling and [^11^C]UCB-J V_T_ showed a very close correlation between V_T_ and synaptophysin immunofluorescence [47], providing cross-validation between the PET measure and one of the most widely used ex vivo synaptic markers (Fig. 2). While promising, this study was performed only on a single animal. Only one other similar study has been performed, which examined relationships between SV2A autoradiography using [^3^H]UCB-J and synaptophysin mRNA levels, finding no correlation [48], however these authors did not look at synaptophysin protein levels. These issues, among others, are discussed in the Limitations section.

Another important issue is whether SV2A PET measures are affected by synaptic activity. To investigate this, Smart et al. [49] studied [^11^C]UCB-J V_T_ and BP_ND_ in 7 healthy volunteers during visual stimulation, finding that, while influx of the tracer to visual cortex correlated with the functional magnetic resonance imaging (fMRI) blood oxygen-level dependent (BOLD) response, there was no change to [^11^C]UCB-J V_T_ or BP_ND_, indicating that these are likely stable measures and independent of acute changes in cortical activity.

In conclusion, of the four PET tracers currently used in clinical research which target SV2A, [^11^C]UCB-J and [^18^F]SynVesT-1 show the best properties. The following sections consider human findings using these tracers and their implications for understanding neuropsychiatric disorders.

## SV2A findings in vivo

### Healthy aging

Five cross-sectional studies have used [^11^C]UCB-J PET to investigate relationships between SV2A levels and age in healthy participants (Table 3, Supplementary Table 2). Studies using V_T_ as an outcome consistently show lower cortical tracer uptake correlating with increasing age, particularly in the caudate nucleus [50, 51] and prefrontal cortex (PFC) [52]. As Table 3 shows, findings are less clear cut where SUVR is the outcome, with smaller [53] or non-significant [54] effects, although studies generally show lower values in older age. Complicating interpretation of the SUVR studies is the finding of a positive correlation between [^11^C]UCB-J SUV in the centrum semiovale (CSO) and age (Table 3), which may lead to underestimation of tracer binding in the rest of the brain in older participants where CSO is used as a reference region. This, along with other limitations of SUVR as a semi-quantitative outcome measure (Box 1, Limitations Section), may explain why changes are not consistently seen in SUVR studies (Table 3). Further work is needed to clarify these findings in SUVR studies, and longitudinal studies would be useful to identify within-subject changes in PET measures over time.

**Table 3:** Results from studies of SV2A PET in healthy participants from studiesshowing reductions in SV2A measures associated ageing.

Diagnosis	Reference	Comparison	Significant differences in SV2A PET measures (% difference, effect size or correlation coefficient)
**HC**	Andersen [98]	Old vs young SUVR	Nil significant
	Fang [52]	Correlation of age with SUVR	Greater age correlated with lower SUVR in mPFC (r = −0.37)
	Mansur [50, 142]	Correlation of age with V_T_/*f*_*p*_	Greater age correlated with lower V_T_/*f*_*p*_ in CN (ΔV_T_/*f*_*p*_ −1.31/year)
	Michiels [53]	Old vs young SUVR	↓ CN (1.7% RPD), ↓ CS
	Toyonaga [51]	HC (age-related) BP_F_	↓ CN (3.6% RPD), ↓ CS (2.1% RPD), ↓ mOC (3.4% RPD)

One study of healthy controls also compared SV2A measures in escitalopram vs placebo (see supplementary Table S2), however is not included here as no significant differences were reported. If both PVC and non-PVC results were available, then only PVC were reported in the table. ↓=lower SV2A PET value in the region indicated. Nonsignificant findings are not reported, but full data can be accessed here GitHub.

*r* correlation coefficient, *BPf* free binding potential, *CN* caudate nucleus, *CS* centrum semiovale, *m* medial, *OC* occipital cortex, *RPD* reduction per decade, *SUVR* standardised uptake value ratio.

Box 1 Advantages and limitations of different pet outcome measures **SUVR:** SUV is the ratio of tissue radioactivity concentration to injected dose divided by body weight. SUVR, or SUV ratio between an ROI and reference region [7], is generally best suited for metabolic tracers where uptake is almost irreversible. For tracers with reversible kinetics, radioactivity in a limited time-window is sensitive to the tracer clearance from tissue, hence strongly dependent on the start and end of the acquisition. SUVR does not require arterial blood sampling (ABS), long PET scans to acquire dynamic images, or complex modelling. However, it is not quantitative, and because it does not model the tracer input function, regional differences in tracer delivery are unaccounted for, increasing sensitivity to individual differences in tracer kinetics [65]. SUVR also requires a reference region in order to normalise for nonspecific binding, which relies on there being a brain region with certain properties, which may not be the case in synaptic PET imaging (see Limitations).

**V**_**T**_**:** V_T_ is the estimate of the ratio between plasma and tissue radioactivity at equilibrium. V_T_ is obtained through the dynamic measurement of both plasma and tissue radioactive concentrations after a tracer bolus injection and use of a kinetic model, reducing the impact of subject variability in tracer delivery and clearance versus SUVR. A key advantage is that it is quantitative. However, this requires an arterial input function (AIF), increasing subject burden, costs, and analysis complexity. It also requires measurement of the plasma free fraction of tracer, *f*_*p*_, which can introduce additional noise into measurements [142], particularly where *f*_*p*_ differs between subjects. In addition, V_T_ does not differentiate between displaceable and nondisplaceable binding, which may increase noise, particularly where there is between-subject variability in nondisplaceable uptake.

**DVR**: DVR is the ratio of V_T_ between the ROI and a reference region. If the reference region does not express the tracer target, then V_T_ here can be assumed to reflectnonspecific binding and free tracer in the reference region. DVR reduces variability in estimation of the AIFs [142]. However, as with SUVR, this assumes that the reference region is a true reference region, which may not always be the case (see Limitations) [59]. The quality of the reference region has large effects on the accuracy of results. Use of a reference region that exhibits specific binding of tracer will over-correct uptake estimates in the ROI, reducing the accuracy of a DVR study to detect changes.

**BP**_**ND**_**:** BP_ND_ is calculated using the ratio of specific to nondisplaceable volume of distribution [21]. The major advantages of BP_ND_ include its versatility, as it can be calculated both using an AIF from V_T_ estimates, providing an outcome measure equalling DVR-1 [7], or using a simplified reference tissue model (SRTM) which is used to estimate the input function from the reference without ABS [161]. The former approach carries the same limitations as DVR. However the latter approach may slightly underestimate uptake compared to DVR [28], and relies on several assumptions which can bias BP_ND_ results in either direction while misleadingly retaining a good fit for the model if not met [59].

### Schizophrenia

Four [^11^C]UCB-J PET studies have been performed in people with schizophrenia (Table 4, Supplementary Table 3) [55–58]. The two studies in patients with chronic illness, using V_T_ [55] and BP_ND_, derived from V_T_ estimates [58] respectively, both showed lower [^11^C]UCB-J uptake in the cortex and hippocampus, with similar effect sizes. In contrast, findings early in the course of illness were not so consistent. Yoon et al. [57] found significantly lower [^11^C]UCB-J BP_ND_, derived from reference tissue estimates, in several cortical and subcortical regions (Table 4) relative to controls, while Onwordi et al. [56], found no significant difference in V_T_ but did find significantly lower [^11^C]UCB-J DVR in the temporal lobe versus controls. Significant negative correlations between frontal tracer uptake and positive symptoms were found in two of the four studies, one each studying early-illness [57] and chronic [58] cohorts, with a similar trend detected in Onwordi’s study investigating early-illness [56] (Table 7).

**Table 4:** Results from studies of SV2A PET in psychiatric illnesses.

Diagnosis	Reference	Comparison	Significant differences in SV2A PET measures (% difference, effect size)
**Addictions**	Angarita [68]	CoUD vs HC BP_ND_	↓ ACC, ↓ vmPFC, ↓ mOFC
		CoUD vs HC V_T_/fp	↓ ACC (d = 0.9), ↓ vmPFC (d = 0.83), ↓ mOFC (d = 0.75)
	D’Souza [69]	CaUD vs HC BP_ND_	↓ Hip (−10.00%, d = 1.2)
	Hou [70]	IGD vs HC SUVR	↓ LN L, ↓ RO, ↓ ACC
**Mood disorders**	Holmes [64]	MDD/PTSD vs HC V_T_	↓ dlPFC (−14.94%, d = 1.14), ↓ ACC (−15.76%, d = 1.3), ↓ Hip (−15.14%, d = 1.1), ↓ CBL (−14.17%, d = 1.54), ↓ FC (−15.28%, d = 1.18), ↓ OC (−14.08%, d = 1.12), ↓ PC (−14.66%, d = 1.03), ↓ Put (−13.67%, d = 1.18), ↓ TC (−14.38%, d = 1.21)
	Casteele [63]	Later life MDD vs HC SUVR	NS
	Holmes [66]	MDD/PTSD vs HC V_T_	↓ dlPFC, ↓ ACC, ↓ Hip
		Low SV2A MDD/PTSD vs high SV2A post ketamine V_T_	↑ dlPFC (8.70%, d = 1.1), ↑ ACC (10.40%, d = 1.2)
**SZ**	Onwordi [55]	SZ vs HC V_T_	↓ FC (d = 0.8), ↓ ACC (d = 0.9), ↓ dlPFC (d = 0.9), ↓ TL (d = 0.9), ↓ OL (d = 0.8), ↓ PL (d = 0.7), ↓ Thal (d = 0.8), ↓ Amg (d = 0.7)
		SZ vs HC DVR	↓ FC (d = 1), ↓ ACC (d = 1), ↓ dlPFC (d = 1), ↓ TL (d = 1.1), ↓ OL (d = 0.9), ↓ PL (d = 0.9), ↓ Thal (d = 0.9)
	Onwordi [108]	SZ vs HC DVR	↓ ACC (d = 0.8)
		SZ vs HC V_T_	↓ ACC (d = 0.9)
	Onwordi [56]	SZ vs HC V_T_ /fp	↓ TL (d = 0.7)
	Radhakrishnan [58]	SZ vs HC BP_ND_	↓ FC (−10.00%, d = 1.01), ↓ ACC (−11.00%, d = 1.24), ↓ Hip (−15.00%, d = 1.29), ↓ OC (−14.00%, d = 1.34), ↓ PC (−10.00%, d = 0.03), ↓ TC (−11.00%, d = 1.23), ↓ Amg (−10.10%, d = 0.91), ↓ FG (−11.10%, d = 1.17), ↓ IC (−11.00%, d = 1.09), ↓ Pallidum (−11.60%, d = 0.98), ↓ Put (−8.10%, d = 0.83), ↓ Thal (−10.90%, d = 1), ↓ VS (−8.50%, d = 0.9)
		SZ vs HC V_T_	↓ ACC (−8.10%, d = 0.87), ↓ Hip (−9.60%, d = 1.24), ↓ OC (−10.50%, d = 1.04), ↓ TC (−8.00%, d = 0.87)
	Yoon [57]	SZ vs HC BP_ND_	↓ Amg L (−21.30%, d = 1.555), ↓ Pars triangularis R (−21.50%, d = 1.488), ↓ FP L (−21.60%, d = 1.485), ↓ Thal R (−17.00%, d = 1.454), ↓ Rost mFG R (−18.50%, d = 1.448), ↓ mOFC R (−18.40%, d = 1.446), ↓ lOFC R (−18.30%, d = 1.446), ↓ Hip R (−17.00%, d = 1.429), ↓ Put L (−16.90%, d = 1.425), ↓ Hip L (−18.20%, d = 1.421), ↓ HG R (−18.50%, d = 1.42), ↓ Rost mFG R (18.5%, d = 1.448), ↓ Hip R (17.00%, d = 1.429), ↓ Put L (16.9%, d = 1.425), ↓ Hip L (18.2%, d = 1.421), ↓ Heschl’s R (18.50%, d = 1.42), ↓ sTG R (18.5%, d = 1.351), ↓ Caud mFG R (19.3%, d = 1.337), ↓ Put R (15.7%, d = 1.33)

If both PVC and non-PVC results were available, then only PVC were reported in the table. Nonsignificant findings are not reported, but full data can be accessed here GitHub.

↓ lower SV2A PET value in the region indicated, *ACC* anterior cingulate cortex, *Amg* amygdala, *BPnd* nondisplaceable binding potential, *CaUD* cannabis use disorder, *CBL* cerebellum, *CoUD* cocaine use disorder, *DVR* distribution volume ratio, *FG* frontal gyrus, *FP* frontal pole, *Hip* hippocampus, *i* inferior, *IC* insular cortex, *IGD* internet gaming disorder, *l* left, *LN* lenticular nucleus, *m* medial/middle, *MDD* major depressive disorder, *NR* not reported, *OC* occipital cortex, *OFC* orbitofrontal cortex, *OL* occipital lobe, *PC* parietal cortex, *PL* parietal lobe, *Put* putamen, *r* right, *RO* Rolandic operculum, *s* superior, *SUVR* standardised uptake value ratio, *SZ* schizophrenia, *TC* temporal cortex, *TG* temporal gyrus, *Thal* thalamus, *(vm/dlp)FC* (ventromedial/dorsolateral pre)frontal cortex, *Vt* volume of distribution.

The use of outcome measures differed between the studies. Onwordi et al. used V_T_ measures and derived DVR using them (see Supplementary), while Yoon et al. used BP_ND_ derived from the SRTM, without ABS. In the latter case, the CSO was used as a reference region, which may violate two of the assumptions required for the model [59], namely that the reference region is devoid of specific binding, and that the CSO and regions of interest have the same nondisplaceable uptake (see Limitations). Both of these violations have been shown in computational models to negatively bias BP_ND_ results, with a greater negative bias at lower BP_ND_ [59], which could thus inflate the difference in measured BP_ND_ between patients and controls. Given this inconsistency and the possibility that differences may be inflated using BP_ND_, further studies reporting V_T_ as well as other outcome measures are needed in early course patients.

Another consideration is that, with the exception of the study by Onwordi et al. (2023), most of the patients in these studies were taking antipsychotic medication. However, administration of the antipsychotics haloperidol and olanzapine to rats for 28 days resulted in no difference in SV2A on markers [55, 60] relative to vehicle-treated rats. Similar results were found on in vitro measures such as neuroligin puncta density [60] and synaptophysin immunoreactivity [61]. In contrast, synaptic density, as observed with microscopy, has been found to be higher with olanzapine and lower with haloperidol in a rat study [62]. Notwithstanding this finding, none of the PET studies have found correlations with current or past antipsychotic use [55–58]. Overall, whilst the present data cannot exclude an effect of antipsychotics on SV2A PET outcomes, it seems unlikely that antipsychotic treatment is having a major effect on them.

### Major depression

Three PET studies have been performed investigating SV2A in major depressive disorder (MDD) (Table 4, Supplementary Table 3). These studies report diverging results, with Casteele et al. [63] showing no change in [^11^C]UCB-J SUVR in later-life depression versus controls, while in a study including people with either MDD or posttraumatic stress disorder (PTSD), Holmes et al. [64] found lower [^11^C]UCB-J V_T_ in the dorsolateral prefrontal cortex (dlPFC), hippocampus and anterior cingulate cortex (ACC) in patients with more severe depressive symptoms compared to controls. The use of different outcome measures may explain the divergence; SUVR studies are more sensitive to noise and inter-individual variability in tracer uptake than quantitative outcome measures using ABS, such as V_T_ (Box 1, Supplementary), and may be expected to require larger sample sizes to detect an effect [65]. In a separate study investigating treatment effects, Holmes et al. found that participants with low V_T_ showed significant increases in [^11^C]UCB-J V_T_ following ketamine in the dlPFC and ACC *(*Table 4), which correlated with a greater reduction in depressive symptoms [66]. A caveat of this post-hoc analysis is that it could reflect regression to the mean. See the review on imaging findings in MDD in this issue of *Neuropsychopharmacology* for further discussion.

Holmes et al. also found that depressive symptoms negatively correlated with [^11^C]UCB-J V_T_ in these three regions (Table 7), including in patients only with a PTSD diagnosis [64]. This was not found in a similar study by Asch et al. [67], who found no correlations between depressive symptomatology and [^11^C]UCB-J V_T_ in subjects with obesity and various psychiatric diagnoses. This study did, however, find significant correlations between anxiety ratings and [^11^C]UCB-J V_T_ in prefrontal regions in all patients (Table 7). Unfortunately, no symptom correlations were explored in the later-life depression study [63]. These findings suggest that subclinical mood and anxiety symptoms may be associated with lower SV2A in the frontal cortex in patients across a range of different diagnoses, although current evidence is limited.

### Substance use disorders and other addictions

Three studies have performed SV2A PET in addictions, using [^11^C]UCB-J V_T_/*f*_p_ in cocaine use disorder [68], [^11^C]UCB-J BP_ND_ in cannabis use disorder [69], and [^18^F]SynVesT-1 SUVR in gaming disorder [70] (Table 4, Supplementary Table 3). Each found lower tracer uptake in patients versus controls, but in mostly non-overlapping regions (Table 4). Only the ACC was implicated in more than one study, showing lower tracer uptake in cocaine use disorder and gaming disorder. Other regions showed lower tracer uptake in only one study: the PFC in cocaine use [68], the hippocampus in cannabis use [69], and the putamen in gaming disorder [70]. Relationships between tracer uptake in these regions with frequency of engagement with the addictive behaviour similarly varied, from significant negative correlations in gaming disorder, to no relationship in cannabis misuse, to significant positive correlations in cocaine misuse (Table 7). The latter finding is paradoxical, given the lower SV2A PET measures in the patient group. The authors suggest it is related to the formation of silent synapses in the brain during cocaine use, which has been observed in mice [71, 72]. They hypothesised that these synapses would be pruned after a period of abstinence, explaining the positive correlation with cocaine consumption. While a general pathophysiology underlying different addictions has been proposed [73], evidence from these three studies does not suggest there is a common pattern of synaptic terminal loss in specific brain regions based on the limited literature to date.

### Alzheimer’s dementia

16 studies have used SV2A PET imaging in AD; all finding lower SV2A PET measures in people with AD relative to controls, although several of these had overlapping samples (Supplementary Table 4). Findings consistent with lower SV2A levels in entorhinal cortex [74–77] and the hippocampus [28, 74, 76–83] (Table 5, Supplementary Table 4) were most widely reported. Studies also report changes in the amygdala [76, 77], the parahippocampus [76, 78], and the thalamus [74, 78, 81] (Table 5). In individuals with mild cognitive impairment (MCI) lower [^11^C]-UCB-J SUVR was found in medial temporal lobe and hippocampus relative to controls [80]. When the same cohort was scanned after 2 years, a small decrease in [^11^C]UCB-J SUVR compared to baseline was found in several cortical regions [79]. In contrast, a longitudinal study in AD found no significant differences in [^11^C]UCB-J DVR versus baseline after 12–18 months [79, 81]. Taken with the findings in MCI, this could suggest that the early stages of AD are characterised by larger longitudinal reductions in SV2A levels than later stages of AD, but further longitudinal studies are needed to test this. O’Dell et al. [84] investigated relationships with amyloid-beta (Aβ), finding a negative correlation between [^11^C]PiB DVR, a marker of Aβ deposition, and hippocampal [^11^C]UCB-J DVR in a cohort of people with MCI AD (*r* = −0.55, *p* = 0.04), but not in an mild AD dementia cohort (*r* = 0.05, *p* = 0.82). These findings suggest that Aβ could be contributing to synaptic degeneration more at the MCI than the dementia stage, when Aβ levels tend to plateau [84].

**Table 5:** Results from studies of SV2A PET in neurodegenerative disorders.

Diagnosis	Reference	Comparison	Significant differences in SV2A PET measures (% difference, effect size, where reported)
**AD/MCI**	Bastin [78]	AD vs HC (V_T_)	↓ Basal forebrain (−37.30%, d = 0.67), ↓ Hip (−30.90%, d = 1.24), ↓ OC (−17.20%, d = 0.65), ↓ PC (−17.60%, d = 0.69), ↓ pFC (−14.30%, d = 0.63), ↓ PPHG (15.60%, d = 0.71), ↓ TC (−14.90%, d = 0.65), ↓ Thal (−15.90%, d = 0.73)
	Chen [74]	AD vs HC (BP_ND_)	↓ EC, ↓ Hip (−44.00%)
		AD vs HC (V_T_)	↓ EC, ↓ Hip (−28.00%), ↓ Pulvinar
	Chen [76]	AD vs HC (DVR)	↓ AG (8.70%, d = 0.91), ↓ Hip (24.80%, d = 1.93), ↓ PC (8.50%, d = 0.84), ↓ PHG (15.00%, d = 1.44), ↓ Pulvinar (18.30%, d = 1.55), ↓ TC (7.60%, d = 1.04), ↓ Thal (12.40%, d = 1.28)
		AD vs HC (DVR)	↓ Amg (17.10%, d = 1.25), ↓ EC (23.20%, d = 1.62)
	Lu [75]	AD vs HC (BP_ND_)	↓ EC, ↓ Hip
	Mecca [77]	AD vs HC (BP_ND_)	↓ Hip (20.00%)
	Tuncel [28]	AD vs HC (BP_ND_)	↓Hip (20.9%), ↓ MTL (17.3%), ↓ GM (16.9%)
	Vanderlinden [79]	aMCI Δ2 years (SUVR)	↓ CC (−6.80%), ↓ FC (−6.60%), ↓ Hip (−7.70%), ↓ Lateral TC (−6.30%), ↓ MsTC (−9.10%), ↓ OC (−6.00%), ↓ PC (−6.80%)
	Vanhaute [80]	MCI vs HC (SUVR)	↓ Hip (19.00%), ↓ TL (17.00%)
	Venkataraman [81]	AD vs MCI (DVR)	↓ CN (25.00%), ↓ Hip (24.00%), ↓ Thal (19.00%)
	Zhang [82]	AD vs HC (SUVR)	↓ FG L, ↓ Hip L, ↓ Hip R, ↓ IC L, ↓ IC R, ↓ iPG R, ↓ mFG, ↓ mFG L, ↓ PCG L, ↓ PoCG R, ↓ sFG L, ↓ sFG R
		AD vs MCI (SUVR)	↓ IC R, ↓ mFG L, ↓ mFG R
**Other Primary Tauopathy**	Holland [86]	CBD vs HC, PSP vs HC (BP_ND_)	↓ Amg (20%, 19%), ↓ CBL (18%, 21%), ↓ CG (15%, 19%), ↓ CN (17%, 21%), ↓ FL (12%, 16%), ↓ Hip (22%, 22%), ↓ Ins (19%, 21%), ↓ Medulla (40%, 55%), ↓ Midbrain (17%, 30%), ↓ NAcc (8%, 15%), ↓ OL (11%, 17%), ↓ PL (12%, 15%), ↓ Pons (19%, 24%), ↓ Put (14%, 18%), ↓ Thal (20%, 21%), ↓ TL (14%, 19%)
		PSP vs HC (BP_ND_)	↓ Pallidum (33.00%), ↓ SN (38.00%)
	Holland [85]	CBD vs HC, PSP vs HC (BP_ND_)	↓ Amg (d = 0.44 (0.12), 0.43 (0.1)), ↓ CBL (d = 0.40 (0.12), 0.41 (0.1)), ↓ CG (d = 0.37 (0.12), 0.52 (0.1)), ↓ CN (d = 0.48 (0.12), 0.65 (0.1)), ↓ FL (d = 0.31 (0.12), 0.44 (0.1)), ↓ Hip (d = 0.34 (0.12), 0.35 (0.1)), ↓ Ins (d = 0.43 (0.12), 0.50 (0.1)), ↓ Midbrain (d = 0.53 (0.12), 0.90 (0.1)), ↓ OL (d = 0.32 (0.12), 0.44 (0.1)), ↓ PL (d = 0.36 (0.12), 0.42 (0.1)), ↓ Put (d = 0.54 (0.12), 0.71 (0.1)), ↓ SN (d = 0.42 (0.12), 0.73 (0.1)), ↓ Thal (d = 0.72 (0.12), 0.59 (0.1)), ↓ TL (d = 0.34 (0.12), 0.44 (0.1))
		PSP vs HC (BP_ND_)	↓ Medulla (d = 0.30 (0.1)), ↓ NAcc (d = 0.62 (0.1)), ↓ Pallidum (d = 0.62 (0.1))
	Holland [91]	CBD vs HC (BP_ND_)	↓ CBL (mean Z-score −1.6), ↓ CN (mean Z-score −1.6), ↓ FL (mean Z-score −1.6), ↓ PL (mean Z-score −1.8), ↓ Put (mean Z-score −1.5), ↓ Thal (mean Z-score −1.8)
		PSP vs HC (BP_ND_)	↓ CC (mean Z-score −1.6), ↓ CN (mean Z-score −2.1), ↓ FL (mean Z-score −1.6), ↓ Midbrain (mean Z-score −1.6), ↓ Pallidum (mean Z-score −1.9), ↓ Thal (mean Z-score −1.6)
		ΔUCB-J PSP/CBD 1 y followup (BP_ND_)	↓ CN (−3.90%), ↓ Presubgenual FC (−3.50%)
	Whiteside [90]	PSP + CBS + bvFTD vs HC (BP_ND_)	↓ Widespread
	Malpetti [89]	bvFTD vs HC (BP_ND_)	↓ Amg L (24.00%, d = −1.48), ↓ Amg R (37.00%, d = −2.09), ↓ CC L (23.00%, d = −2.00), ↓ CC R (24.00%, d = −2.26), ↓ FC L (23.00%, d = −2.33), ↓ FC R (21.00%, d = −2.33), ↓ Hip L (26.00%, d = −1.14), ↓ Hip R (36.00%, d = −1.96), ↓ IC L (28.00%, d = −2.00), ↓ IC R (33.00%, d = −2.68), ↓ OC L (12.00%, d = −1.14), ↓ OC R (13.00%, d = −1.23), ↓ PC L (16.00%, d = −1.89), ↓ PC R (16.00%, d = −1.81), ↓ TC L (24.00%, d = −2.28), ↓ TC R (23.00%, d = −2.17), ↓ Thal L (26.00%, d = −1.80), ↓ Thal R (35.00%, d = −1.67)
**α-synucleinopathy**	Andersen [98]	DLB/PDD vs HC (SUVR)	↓ iPC, ↓ lateral OC, ↓ lateral OTC, ↓ M1S1, ↓ mFG, ↓ mOC, ↓ OFC, ↓ SN, ↓ sPL
		nPD vs HC (SUVR)	↓ SN
	Andersen [96]	DLB/PDD vs HC (SUVR)	↓ CBL (−26.00%), ↓ FC (−21.00%), ↓ IC (−46.00%), ↓ mTC (−20.00%), ↓ OC (−28.00%), ↓ PC (−25.00%), ↓ TC L (−21.00%), ↓ Thal (−22.00%)
	Delva [94]	PD vs HC (SUVR)	↓ SN (−14.40%)
	Delva [101]	PD vs HC (SUVR)	↓ SN (15.70%)
	Matuskey [93]	PD vs HC (BP_ND_)	↓ Locus coeruleus (17.00%), ↓ OFC (11.00%), ↓ PCC (15.00%), ↓ PHG (12.00%), ↓ Red nucleus (31.00%), ↓ SN (45.00%), ↓ vmPFC (11.00%)
	Nicastro [104]	LBD vs HC (BP_ND_)	↓ Cuneus, ↓ iFG, ↓ mFG, ↓ OCC, ↓ PCG, ↓ PL, ↓ sFG, ↓ sPC, ↓ sTC, ↓ TL
	Wilson [95]	PD vs HC (V_T_)	↓ BS (9.40%, d = 0.81), ↓ CN (15.00%, d = 0.82), ↓ DRaphe (9.00%, d = 0.83), ↓ FC (10.30%, d = 0.86), ↓ Ins (7.90%, d = 0.77), ↓ OC (11.00%, d = 1), ↓ PC (11.00%, d = 0.88), ↓ Put (9.70%, d = 0.99), ↓ TC (9.00%, d = 0.92), ↓ Thal (11.60%, d = 0.74)
**HD**	Delva [97]	HD vs HC (SUVR)	↓ CBL (11% ( ± 9%)), ↓ CN (25% ( ± 14%)), ↓ FC (8% ( ± 8%)), ↓ OC (9% ( ± 8%)), ↓ Pallidum (24% ( ± 15%)), ↓ PC (9% ( ± 8%)), ↓ Put (28% ( ± 13%)), ↓ TC (9% ( ± 9%))
		mHD vs HC (SUVR)	↓ CBL (−14.00%), ↓ CN (−31.00%), ↓ FC (−11.00%), ↓ GM (−12.00%), ↓ Pallidum (−30.00%), ↓ PC (−11.00%), ↓ Put (−33.00%), ↓ TC (−12.00%)
		pmHD vs HC (SUVR)	↓ CN (−16.00%), ↓ Put (−19.00%)
	Delva [102]	ΔHD (Y0 vs Y2), ΔHC (Y0 vs Y2) (SUVR)	↓ CN (−4.5%, −0.8%), ↓ Put (−3.6%, −0.1%)
		ΔpmHD (Y0 vs Y2), ΔmHD (Y0 vs Y2) (SUVR)	↓ CN (−2.9%, −5.4%), ↓ Pallidum (−0.8%, −4.2%)
**SCA**	Chen [162]	Ataxic vs. HC (SUVR)	↓ CBL L (−11.43%), ↓ CBL R (−11.71%), ↓ CN L (−8.67%), ↓ CN R (−7.42%), ↓ Medulla (−8.33%), ↓ Midbrain (−7.61%), ↓ OC L (−4.95%), ↓ OC R (−4.94%), ↓ Pons (−8.03%), ↓ Put L (−3.96%), ↓ Put R (−3.55%), ↓ Vermis (−15.95%)
		Ataxic vs. pre-ataxic (SUVR)	↓ CBL L (−8.22%), ↓ CBL R (−7.69%), ↓ CN L (−7.33%), ↓ CN R (−5.60%), ↓ Medulla (−10.60%), ↓ Midbrain (−5.21%), ↓ OC L (−3.52%), ↓ OC R (−3.68%), ↓ Pons (−8.03%), ↓ Put L (−3.15%), ↓ Put R (−3.77%), ↓ Vermis (−10.75%)
		Pre-ataxic vs HC (SUVR)	↓ Vermis (not significant after PVC) (−5.83%)
**TLE**	Finnema [116]	Asymmetry in BP_ND_ in TLE vs HC	Amg (7% ( ± 6%), 3% ( ± 5%)), EC (3% ( ± 14%), 9% ( ± 6)), Fusiform (1% ( ± 6%), 8% ( ± 4)), Hip (17% ( ± 5%), 0% ( ± 4)), Ins (2% ( ± 4%), 10% ( ± 4)), ParaHip (0% ( ± 5%), 9% ( ± 6)), TC (1% ( ± 5%), 9% ( ± 4)), Thal (4% ( ± 6%), −1% ( ± 2))

If both PVC and non-PVC results were available, then only PVC were reported in the table. Nonsignificant findings are not reported, but full data can be accessed here GitHub.

↓ lower SV2A PET value in the region indicated, *AD* Alzheimer’s dementia, *AG* angular gyrus, *Amg* amygdala, *BPnd* nondisplaceable binding potential, *BS* brainstem, *(bv)FTD* (behavioural variant) frontotemporal dementia, *CBD* corticobasal degeneration, *CBL* cerebellum, *CG* cingulate gyrus, *CN* caudate nucleus, *DRaphe* dorsal raphe, *DVR* distribution volume ratio, *EC* entorhinal cortex, *FG* frontal gyrus, *FL* frontal lobe, *GM* grey matter, *Hip* hippocampus, *i* inferior, *IC* insular cortex, *l* left, *m* medial/middle, *LBD* Lewy body dementia, *M1S1* primary sensorimotor cortex, *MCI* mild cognitive impairment, *MsTC* mesotemporal cortex, *NAcc* nucleus accumbens, *NR* not reported, *OC* occipital cortex, *OFC* orbitofrontal cortex, *OL* occipital lobe, *OTC* occipitotemporal cortex, *PC* parietal cortex, *PCC* posterior cingulate cortex, *PCG* posterior cingulate gyrus, *PD* Parkinson’s disease, *(pm)HD* (premanifest) Huntington’s disease, *PoCG* postcentral gyrus, *PL* parietal lobe, *PHG* parahippocampal gyrus, *PSP* progressive supranuclear palsy, *Put* putamen, *PVC* partial volume correction, *r* right, *s* superior, *SCA* spinocerebellar ataxia, *SN* substantia nigra, *SUVR* standardised uptake value ratio, *Thal* thalamus, *TLE* temporal lobe epilepsy, *(vm/dlp)FC* (ventromedial/dorsolateral pre)frontal cortex, *Vt* volume of distribution.

### Other primary tauopathies

Six cross-sectional studies have been published SV2A PET in non-AD tauopathies [85–90] (Table 5, Supplementary Table 4). Studies of progressive supranuclear palsy (PSP) and corticobasal degeneration (CBD) have consistently shown lower BP_ND_ with [^11^C]UCB-J [85, 87, 90, 91], affecting most cortical and subcortical regions in CBD, and all regions reported in PSP (Table 5). Effect sizes for lower BP_ND_ versus controls in PSP were generally larger in the basal ganglia compared to CBD [85, 86]. A further two studies have reported findings specifically in behavioural variant frontotemporal dementia (bvFTD) [88, 89]. Salmon et al. showed lower [^18^F]UCB-H V_T_ in the parahippocampal gyrus only, which did not survive family-wise-error correction, while Malpetti et al. reported lower [^11^C]UCB-J BP_ND_ in widespread cortical, but not subcortical, regions. The less extensive alterations seen in Salmon et al. could reflect the fact that their sample showed relatively milder impairments on the Mini Mental State Exam (MMSE) (patients = 25.4, controls = 29.1) than those in the study by Malpetti et al. (MMSE score: patients = 22.3, controls = 29.5). A further study investigated [^11^C]UCB-J BP_ND_ in a sample that included patients with PSP, bvFTD or CBD [90]. They found globally lower BP_ND_ in the combined clinical group versus controls in cortical and subcortical regions in a voxel-wise analysis. Independent component analysis found more marked cortical SV2A changes in bvFTD than the other conditions. The component incorporating the medial parietal and frontal lobe showed lower BP_ND_ in CBD over PSP (*p* = 0.024), although no components showed lower BP_ND_ in PSP compared to the other clinical groups. These findings in total are broadly consistent with the clinical spectrum of tauopathies [92], with PSP showing greater pathology in subcortical nuclei, bvFTD more cortical pathology, and CBD showing similar involvement of both cortical and subcortical regions.

One study in CBD and PSP has investigated changes in synaptic measures over time. Holland et al. [91] found a significant overall decline in [^11^C]UCB-J BP_ND_ over 1 year, particularly in the right caudate and left frontal cortex (Table 5). Using principal component analysis, the authors also showed that higher loading on a component incorporating the rate of reductions in frontal and cingulate cortical [^11^C]UCB-J BP_ND_ were associated with faster progression of symptoms (*r* = 0.47, *p* = 0.03) and more rapid cognitive decline (*r* = −0.62, *p* = 0.003). Indeed, where this was reported, all cross-sectional studies described here also found significant negative correlations between BP_ND_ and symptom severity, including cognitive dysfunction. This was found in in striatal [90] regions and global GM [86] in PSP/CBD, and cortical regions in bvFTD [89] (Table 7). Overall, with the exception of one study in patients with milder illness, there is convergent evidence that tauopathy is associated with widespread reductions in SV2A PET measures, affecting expected regions based on the clinical syndrome and showing convincing relationships with symptoms.

### α-Synucleinopathies

Four cross-sectional PET studies have been performed in Parkinson’s disease (PD) [93–96] (Table 5 Supplementary Table 4). While all four found lower [^11^C]UCB-J BP_ND_ [93], V_T_ [95] or SUVR [97, 98] in the patient group, there were no specific nuclei or cortical areas showing changes in all four studies (Table 5). The substantia nigra, a key nucleus in the pathophysiology of PD, showed significantly lower tracer uptake in three studies [93, 97, 98], while Wilson et al. [95] found changes in the dorsal raphe nuclei and the brainstem. Other brain regions implicated in each study varied, as did the clinical severity of each study’s sample. Wilson et al. found fairly widespread lower V_T_ in cortical and subcortical regions (Table 5), surprisingly, in a patient group with the mildest symptom severity ratings of all the studies (mean Movement Disorder Society - Unified Parkinson’s Disease Rating Scale III (MDS-UPDRS-III) score 20.1), and with no cognitive impairment. Given only two studies used the same outcome measure, it is difficult to assess whether different analysis methods may explain heterogeneity in the findings across regions or if it could relate to clinical differences, however it is notable that the two which used SUVR both found lower tracer uptake in the substantia nigra only, despite including patients with relatively severe symptoms (MDS-UPDRS-III) score 26.6 [96] and 41.5 [94] respectively).

Two longitudinal studies found no significant change in SV2A PET measures over 10–24 months, despite progression in MDS-UPDRS-III ratings from 24.3 to 27.6 [97] and 23.3 to 31.3 [95] respectively, suggesting that changes detected with SV2A PET are not related to motor progression; only one study [97] reported correlations, but these were nonsignificant. In contrast, uptake of the dopamine transporter (DAT) tracer [18 F]FE-PE2I showed longitudinal decline, which correlated with increasing clinical scores in the putamen (*r* = −0.53) and dorsal striatum (*r* = −0.51) contralateral to the least affected body side at follow-up.

SV2A imaging in PD does therefore show evidence for lower tracer uptake in expected mesencephalic regions, however the anatomical extent and severity of synaptic protein loss shows little relationship with motor symptoms, except in participants with mild [95] or subclinical [99] disease. Taken with dopaminergic imaging findings that do show relationships with motor symptoms [97], this indicates that SV2A PET is unlikely to provide much clinical value in evaluating motor symptoms of PD. Nevertheless, synaptic alterations may underlie cognitive impairments in PD [100]. Significant negative correlations between tracer uptake and cognitive performance were found in patients with Lewy body dementia (LBD) and PD dementia by Andersen et al., but only Matuskey et al. analysed relationships between BP_ND_ and cognitive function in people with PD without dementia. They found no significant correlations between cognitive scores and tracer uptake, but their analysis was restricted to SV2A measures in subcortical areas and motor cortex. It would be useful for future studies to investigate relationships in cortical regions which may be more closely implicated to cognitive impairments.

### Huntington’s disease

Two SV2A PET studies have been conducted in Huntington’s disease (HD); one cross-sectional [101] and the second its longitudinal extension [102] (Table 5, Supplementary Table 4). Delva et al. [101] measured [^11^C]UCB-J SUVR in 18 patients with HD, 7 of whom had premanifest disease (i.e., not showing motor signs), compared to 15 controls. [^11^C]UCB-J uptake was significantly lower in total GM in the manifest group versus controls, as well as in widespread cortical, cerebellar and subcortical regions (Table 5). Voxelwise analysis showed the most marked differences were in the bilateral striatum and thalamus. The premanifest group showed lower [^11^C]UCB-J SUVR versus controls in the putamen and caudate nucleus only (Table 5). [^11^C]UCB-J SUVR-1 in the caudate was significantly correlated with more severe motor symptoms (Table 7), indicating that the motor symptoms of HD are related to synaptic changes in striatal regions. Repeated measures after a 2-year interval found that the premanifest group had progressed to show similar regional reductions in the SV2A marker as the manifest group when compared to controls. However, change in symptoms in the whole cohort did not correlate with changes in SUVR. Notwithstanding this, the HD findings suggest lower SV2A is localised to striatal regions before motor symptom onset in HD, and spreads to include much of the brain’s GM in manifest disease.

### Relationships between SV2A PET measures and magnetic resonance imaging

The relationship between SV2A PET measures and GM volume (GMV) measured using MRI was reported in nine studies [55, 58, 63, 68, 77, 83, 88, 103, 104]. While studies frequently found GM atrophy across disorders, only two found positive correlations between GMV and SV2A PET tracer uptake in patients [83, 88], with five studies finding no relationships [55, 58, 63, 68, 104] (Table 6). In tauopathies, illnesses strongly associated with brain atrophy [105], lower GMV was anatomically less extensive than lower SV2A tracer uptake [77, 90] and showed smaller effect sizes in group comparisons [87], suggesting that SV2A changes may precede detectable GM atrophy in neurodegeneration. One study also reported that GMV showed weaker correlations with cognitive dysfunction than did SV2A tracer uptake [103]. This could suggest that the GMV signal is less closely related to the pathophysiological process underlying symptoms than SV2A measures [106].

**Table 6:** Results from correlations with other imaging measures.

Imaging Modality	Sample	Regional SV2A	Finding	Study
**GMV**	**CoUD**	ACC V_T_/fp	*r* = −0.16 (NS)	Angarita [68]
		VS V_T_ /fp	*r* = 0.19 (NS)	
		lOFC V_T_ /fp	*r* = −0.01 (NS)	
		mOFC V_T_ /fp	*r* = 0.37 (NS)	
		vmPFC V_T_ /fp	*r* = −0.06 (NS)	
	**MDD**	Hip NR	NR (NS)	Casteele [63]
		TC NR	NR (NS)	
		pFC NR	NR (NS)	
	**AD**	Fusiform L V_T_	*r* = 0.45	Moallemian [83]
		Hip L V_T_	*r* = 0.47	
		Hip R V_T_	*r* = 0.19 (NS)	
		PHG R V_T_	*r* = 0.22 (NS)	
		TC L V_T_	*r* = −0.01(NS)	
	**LBD**	Global BP_ND_	NR (NS)	Nicastro [104]
	**SZ**	ACC V_T_	*r* = 0.19 (NS)	Onwordi [55]
		FC V_T_	*r* = 0.20 (NS)	
		Hip V_T_	*r* = 0.21 (NS)	
	**SZ**	ACC BP_ND_	*r* = −0.14 (NS)	Radhakrishnan [58]
		FC BP_ND_	*r* = 0.−0.01(NS)	
		OC BP_ND_	*r* = 0.14 (NS)	
		PC BP_ND_	*r* = 0.49 (NS)	
		TC BP_ND_	*r* = −0.22 (NS)	
		mTC V_T_	*r* = 0.70	Salmon [88]
**NDI**	**AD** + **HC**	FL DVR	*R*^2^ = 0.17	Venkataraman [81]
		PL DVR	*R*^2^ = 0.33	
**ODI**	**PSP, CBS**	Widespread	NR	Mak [87]
	**HC**	Widespread	NR	
	**AD** + **HC**	PL DVR	*R*^2^ = 0.15	Venkataraman [81]
		CN DVR	*R*^2^ = 0.20	
	**PSP, CBS, bvFTD**	Global BP_ND_	*β* = 0.54	Whiteside [90]
	**HC**	Global BP_ND_	*β* = 0.39	
**MRS** (ACC Glu/Cr)	**SZ**	ACC DVR	*r* = 0.32 (NS)	Onwordi [108]
		Hip DVR	*r* = 0.30 (NS)	
**FC**	**HC**	Striatum V_T_	*r* = 0.44	Fang [52]
		mPFC V_T_	*r* = 0.64	
		mPFC V_T_	*r* = 0.36	
		mPFC V_T_	*r* = 0.35	
		Striatum V_T_	*r* = 0.34	
	**PSP, CBD, bvFTD**	Striatal ICA BP_ND_ loading	*β* = 0.4	Whiteside [90]
		Medial PL/FL ICA BP_ND_ loading	*β* = 0.29	
		Left frontoparietal ICA BP_ND_ loading	*β* = 0.27	
		Posterior cingulate ICA BP_ND_ loading	*β* = 0.43	
		Left lateral frontal lobe ICA BP_ND_ loading	*β* = 0.40	
**FC** (lMFG)	**AD** + **MCI**	mFG L SUVR	*r* = 0.73	Zhang [82]
		mFG R SUVR	*r* = 0.61	
**FC** (lMFG-lIFG)	**AD** + **MCI** + **HC**	mFG L SUVR	*r* = 0.67	
		mFG R SUVR	*r* = 0.61	
**FC** (dlPFC-PCC)	**MDD**	dlPFC V_T_	*r* = −0.6	Holmes [64]
**[**^**18**^**F]flortaucipir BP**_**ND**_	**AD**	Multiple ROI BP_ND_	*r* = −0.47	Coomans [111]
**[**^**18**^**F]flortaucipir SUVR** (EC)	**AD** + **HC**	Hip DVR	*r* = −0.61	Mecca [103]
**[**^**18**^**F]AV-1451 BP**_**ND**_ (global)	**PSP, CBS**	Global BP_ND_	*β* = 0.4, *t* = 3.6,	Holland [85]
**[**^**18**^**F]-MK-6240 SUVR** (MTL)	**MCI**	MTL SUVR	*r* = −0.76	Vanhaute [80]
**[**^**18**^**F]-MK-6240 SUVR**	**MCI**	TC, TPC, OC SUV	Negative correlations (voxelwise)	Vanderlinden [79]
**[**^**18**^**F]FDG PET**	**AD**	Hip DVR	*R*^2^ = 0.86	Chen [76]
		Precuneus DVR	*R*^2^ = 0.59	
	**TLE**	Hip BPnd	*R*^2^ = 0.38	Finnema [116]
**[**^**18**^**F]FDG PET** (CN)	**HD**	Global SUVR	*R*^2^ = 0.50	Delva [97]
**[**^**18**^**F]FDG PET** (putamen)		Global SUVR	*R*^2^ = 0.55	
**[**^**11**^**C]PiB DVR** (global)	**AD**	lPC DVR	*r* = 0.03	O’Dell [84]

If both PVC and non-PVC results were available, then only PVC were reported in the table. Nonsignificant findings are not reported, except where relevant to the main text, but full data can be accessed here GitHub.

*AD* Alzheimer’s dementia, *AG* angular gyrus, *Amg* amygdala, *BPnd* nondisplaceable binding potential, *(bv)FTD* (behavioural variant) frontotemporal dementia, *CBD* corticobasal degeneration, *CBL* cerebellum, *CG* cingulate gyrus, *CN* caudate nucleus, *DRaphe* orsal raphe, *DVR* distribution volume ratio, *EC* entorhinal cortex, *FG* frontal gyrus, *FISO* fraction of Gaussian isotropic diffusion, *FL* frontal lobe, *GM(V)* grey matter (volume), *HC* healthy control, *Hip* hippocampus, *i* inferior, *IC* insular cortex, *K1* delivery rate constant, *Ki* inhibition constant, *l* lateral, *L* left, *m* medial/middle, *LBD* Lewy body dementia, *MCI* mild cognitive impairment, *MDD* major depressive disorder, *MTL* medial temporal lobe, *NDI* neurite density index, *NR* not reported, *OC* occipital cortex, *ODI* orientation dispersion index, *OL* occipital lobe, *PC* parietal cortex, *PCC* posterior cingulate cortex, *PCG* posterior cingulate gyrus, *PD* Parkinson’s disease, *(pm)HD* (premanifest) Huntington’s disease, *PL* parietal lobe, *PHG* parahippocampal gyrus, *PSP* progressive supranuclear palsy, *PTSD* posttraumatic stress disorder, *R1* ROI delivery rate constant (K1) normalised to cerebellar K1, *r* right, *ROI* region of interest, *s* superior, *SN* substantia nigra, *SUVR* standardised uptake value ratio, *SV2A* synaptic vesicle glycoprotein 2A, *SZ* schizophrenia, *Thal* thalamus, *TLE* temporal lobe epilepsy, *TPC* temporoparietal cortex, *(vm/dlp)FC* (ventromedial/dorsolateral pre)frontal cortex, *VOI* volume of interest, *Vt* volume of distribution.

Neurite orientation dispersion and density imaging (NODDI) can provide measures of both the density and angular variation of neurites, with the signal dependent on factors such as dendritic arborisation [107]. Orientation dispersion imaging (ODI) has been measured in three tauopathy studies which also reported SV2A PET measures [81, 87, 90]. All three showed positive correlations with SV2A tracer uptake, which were widespread across the brain in two [87, 90] but limited to the parietal lobe and caudate in the third study [81], which also showed positive correlations between BP_ND_ and neurite density (Table 6). Two of these studies [81, 90] additionally detected positive correlations in healthy controls. NODDI is still a novel imaging measure, however, and further work is needed to understand how NODDI findings relate to synaptic loss, and SV2A imaging.

The use of multimodal imaging can also provide information on the loss of specific synapses. Onwordi et al.’s study in schizophrenia [108] investigated correlations between SV2A and regional glutamate concentrations as measured by proton magnetic resonance spectroscopy (MRS). There was a significant positive correlation between SV2A measures and glutamate in the hippocampus and ACC of healthy participants, suggesting that a large proportion of the SV2A PET signal is related to glutamatergic terminals. Interestingly, this relationship was lost in people with schizophrenia. Putatively this could be explained by the depletion of glutamatergic terminals in the condition, which would be anticipated to weaken the normal correlation between SV2A levels and glutamate levels. However, this interpretation remains speculative [109].

Finally, four studies [52, 64, 82, 90] investigated relationships between SV2A PET and functional connectivity in the frontal lobe, as measured by BOLD MRI (Table 6). Fang et al.’s study in healthy controls [52] found significant positive correlations between [^11^C]UCB-J V_T_ in both the medial PFC and striatum and connectivity of the anterior default mode network, measured with fractional amplitude of low-frequency fluctuations, a resting-state MRI measure indexing network connectivity. Whiteside et al.’s analysis [90] combining people with tauopathy and healthy volunteers found significant positive associations between [^11^C]UCB-J BP_ND_ and functional connectivity. Positive correlations in both healthy controls and patients were also found in an AD study [82], where tracer uptake in the middle frontal gyrus correlated with that region’s functional connectivity with the inferior and superior frontal gyri (Table 6). In Holmes et al.’s MDD study [64], dlPFC [^11^C]UCB-J V_T_ was negatively correlated with dlPFC-posterior cingulate functional connectivity in the patient group. The authors suggest that this reflects lower SV2A levels underlying weaker switching between the central executive and default mode networks. Overall, the fMRI findings suggest that SV2A levels in some regions influence the connectivity of neural networks, consistent with a central role for synapses in brain network function.

#### Relationships between SV2A and other PET measures

Five studies [79, 80, 85, 110, 111] compared SV2A and tau accumulation, measured with the PET tracers [^18^F]flortaucipir or [^18^F]AV-1451. SV2A tracer uptake and tau accumulation were negatively correlated in AD [110, 111], as well as in MCI [80] (Table 6), but positively correlated in PSP/CBD [85]. However, the spatial pattern of tau accumulation was more widespread than the regions showing lower [^11^C]UCB-J BP_ND_ or SUVR [80, 111]. At 2 year follow up, tau accumulation and reductions in SV2A tracer uptake in MCI continued to follow this spatial pattern, with tau accumulation remaining more widespread, preceding later changes in SV2A measures [79]. Two studies, in AD [84] and PSP/CBD [90] respectively, reported that at the individual level, lower tau correlates with higher SV2A, but this relationship becomes negative with higher cortical tau, explaining the positive correlation detected by Holland et al. [85]. This may reflect a degree of compensatory upregulation of synapses early in the disorder, which has been reported in mouse models of mutant tau overexpression [112]. These studies are therefore consistent with the theory suggesting that tau accumulation precedes and underlies synaptic pathology in tauopathies [112–115]. This could be tested by determining whether tau PET measures predict the subsequent SV2A reductions.

Three studies have investigated correlations between between FDG and SV2A PET measures, finding significant positive correlations between the measures in AD [76], HD [101] and temporal lobe epilepsy [116] (Table 6). This is expected, considering synapses are responsible for 43–55% of brain adenosine triphosphate (ATP) use [117, 118]. However, interestingly, there is evidence for a greater spatial extent of FDG than SV2A PET alterations, indicating FDG may capture metabolic signs of synaptic impairment before overt loss of synaptic terminals [119]. However, it is important to note FDG changes could also reflect degeneration of the axons, glia and cell bodies, which make up the remaining half of brain ATP use [117].

## Discussion

### Comparison of findings across disorders

SV2A PET studies have shown lower tracer brain uptake in every illness studied to date, although the specific regions involved and the effect size of findings often vary between studies of the same disorders, and there are some inconsistencies. Several factors, including analysis methods, study power, differences in stage or severity of disorder or other sources of heterogeneity, could contribute to inconsistencies between studies. We recommend data sharing as one strategy to address some of these issues, as this would permit the same analysis to be used for all data, boost power, and potentially enable clinical sources of heterogeneity to be investigated. Interestingly, the hippocampus and PFC were implicated in most illnesses studied (Tables 4, 5), with the exception of primary motor disorders. This is consistent with the hippocampus and PFC being regions implicated in a variety of affective, intellectual and motivational processes which are central to illnesses with behavioural and cognitive symptoms [120]; and is consistent with evidence that hippocampal-prefrontal pathways consistently show dysfunction in electrophysiological and neuroimaging studies across multiple psychiatric illnesses [120, 121]. Generalised cortical changes were characteristic of neurodegenerative illnesses associated with dementia, in particular tauopathies, HD, PD dementia and LBD. Subcortical changes were seen in all illnesses with motor symptoms, such as spinocerebellar ataxia, HD, PSP and PD, but also in schizophrenia and MDD, which could reflect the role of these regions in salience and affective states [122]. It should be noted, however, that while disorders may show involvement of the same brain regions, the mechanisms behind altered SV2A may differ between illnesses, and could preferentially affect synapses in specific cortical layers, as may be the case in schizophrenia [123], or of a certain neurotransmitter class.

An important consideration is that SV2A alterations in the disorders we have reviewed may not be causal. They could be the secondary consequences of other brain changes and/or confounders associated with the disorder. For example, neuropsychiatric disorders are often associated with social isolation. Indeed, reduced synaptic density secondary to isolation and reduced environmental richness has been detected in animal models [124], which has measurable effects on cognition [125]. Notwithstanding these considerations, findings that SV2A PET measures in cortical regions are related to functional connectivity measures across the brain highlights that SV2A alterations in specific cortical regions could have widespread effects, and potentially lead to mood [126] and anxiety [127] symptoms, negative symptoms [128], and cognitive impairments [129]. This could account for observations that these symptoms and impairments are seen in many neurological and psychiatric disorders. To date, longitudinal studies have shown decreases in SV2A PET measures and worsening of symptoms in neurodegenerative illnesses, supporting a causal role for synaptic changes in these disorders, but there have not been longitudinal studies in psychiatric disorders. These would be of high value to help determine causal relationships between synaptic alterations and psychiatric outcomes.

### Relationship between SV2A PET measures and cognitive function

24 studies have correlated SV2A PET indices with cognitive measures, with twenty showing significant correlations (Table 7); four did not [93, 97, 98, 111]. In all studies reporting significant findings, lower SV2A PET measures in cortical regions correlated with poorer cognitive function. These covered a range of neuropsychiatric conditions, including mood disorders [67, 130], schizophrenia [57, 58], addictions [69, 70], AD [74, 77–82, 103], amyloid-negative tauopathies [86, 89–91], LBD [98] and HD [101]; this HD study was the only one showing significant correlations between cognition and subcortical tracer uptake, but these did not survive correction for multiple comparisons.

**Table 7:** Results from correlations of regional or global SV2A measures with symptoms and cognitive measures across psychiatric and neurodegenerative diagnoses.

Symptom/ cognitive domain	Diagnosis	Measure	Region	Finding	Study
**Attention**	**SZ**	Detection Test	OC BP_ND_	*r* = −0.66	Radhakrishnan [58]
**Executive function**	**AD**	TMT-B, SCWT	AD-affected ROI DVR	*R*^2^ = 0.30	Mecca [110]
	**IGD**	SSRT	ACC SUVR	*r* = −0.573	Hou [70]
			RO R	*r* = −0.527	
	**LBD**	MoCA executive function domain	mFG SUVR	*R*^2^ = 0.307	Andersen [98]
	**bvFTD**	IFS	CC BP_ND_	*r* = 0.620	Malpetti [89]
			FL BP_ND_	*r* = 0.754	
			PL BP_ND_	*r* = 0.025	
			voxelwise BP_ND_	*r* ≥ 0.8	
**Global cognition**	**AD**	CDR-SB	Hip BP_ND_	*r* = −0.61	Chen [74]
			Hip DVRcb	*r* = −0.62	Mecca [77]
		MMSE	Hip DVR	*r* = 0.77	Vanhaute [80]
			Hip V_T_	*r* = 0.57	Bastin [78]
			TL V_T_	*r* = 0.41	
			pFC V_T_	*r* = 0.40	
		Global Cognition	AD-affected ROI DVR	*R*^2^ = 0.33	Mecca [110]
	**AD** + **MCI**	MMSE	Hip R SUVR	*r* = 0.309	Zhang [82]
			mFG-sFG R connectivity	*r* = 0.289	
	**AD** + **MCI** + **HC**	MMSE	IC R SUVR	*r* = 0.213	
			mFG L SUVR	*r* = 0.124	
			mFG R SUVR	*r* = 0.278	
			mFG-sFG R connectivity	*r* = 0.231	
	**PSP, CBS**	ACE R	global BP_ND_	*r* = 0.52	Holland [86]
		Rate of change in ACE R	Δglobal BP_ND_	*r* = −0.62	Holland [91]
	**SZ**	BACS	HG R	*R*^2^ = 0.339	Yoon [57]
			mFG R	*R*^2^ = 0.286	
			sTG R	*R*^2^ = 0.321	
	**MCI** + **HC**	MMSE	mTL SUVR	*r* = 0.47	Vanderlinden [79]
	**bvFTD**	ACE R	CC BP_ND_	*r* = 0.700	Malpetti [89]
			FL BP_ND_	*r* = 0.791	
			PL BP_ND_	*r* = 0.591	
			voxelwise BP_ND_	*r* ≥ 0.8	
	**PSP, CBS, bvFTD**	ACE-R	Medial PL/FL ICA BP_ND_ loading	*β* = 0.44	Whiteside [90]
			Frontoparietal ICA BP_ND_ loading	*β* = 0.49	
			ACC/IC ICA BP_ND_ loading	*β* = 0.64	
			Lateral FL ICA BP_ND_ loading	*β* = 0.47	
**Language**	**AD**	BNT	AD-affected ROI DVR	*R*^2^ = 0.23	Mecca [110]
		Picture naming & semantic fluency	Hip DVR	*R*^2^ = 0.68	Venkataraman [81]
			PL DVR	*R*^2^ = 0.39	Venkataraman [81]
**Memory**	**AD**	episodic memory	Hip BP_ND_	*r* = 0.56	Chen [74]
**Processing speed**	**AD**	TMT-A, DSS	AD-affected ROI DVR	*R*^2^ = 0.28	Mecca [110]
**Social cogniton**	**SZ**	MATRICS SEC	PCC BP_ND_	*r* = 0.74	Radhakrishnan [58]
		MCCB	FC BP_ND_	*r* = 0.64	
**Verbal memory**	**AD**	RAVLT	Hip DVR	*r* = 0.60	Vanhaute [80]
		RAVLT DR	Hip DVR	*r* = 0.70	
		RAVLT DR	AD-affected ROI DVR	*R*^2^ = 0.24	Mecca [110]
	**AD** + **MCI**	AVLT	IC R SUVR	*r* = 0.279	Zhang [82]
			mFG L SUVR	*r* = 0.259	
			mFG R SUVR	*r* = 0.282	
		BNT	Hip L SUVR	*r* = 0.390	
			Hip R SUVR	*r* = 0.460	
			IC R SUVR	*r* = 0.281	
			mFG L SUVR	*r* = 0.257	
			mFG R SUVR	*r* = 0.262	
		RAVLT	Hip L SUVR	*r* = 0.467	
			Hip R SUVR	*r* = 0.533	
	**CaUD**	RAVLT	Hip BPND	Φ = 0.67	D’Souza [69]
	**LBD**	MoCA (language domain)	OTC SUVR	*R*^2^ = 0.060	Andersen [98]
	**MDD/PTSD**	ISL	ACC V_T_	*r* = 0.41	Holmes [64]
	**MCI** + **HC**	AVF	mTL SUVR	*r* = 0.42	Vanderlinden [79]
		RAVLT	mTL SUVR	*r* = 0.53	
			mTL SUVR	*r* = 0.47	
**Visual attention**	**NWPsy + OWPsy**	Identification Test (response time)	CBL V_T_	*r* = −0.501	Asch [67]
			Hip V_T_	*r* = −0.422	
			OFC V_T_	*r* = −0.420	
			dlPFC V_T_	*r* = −0.439	
			vmPFC V_T_	*r* = −0.444	
**Visuospatial**	**AD**	DMS48	PHG V_T_	*r* = 0.46	Bastin [78]
		WAIS-III block design, WAIS-III picture completion, ROCF	AD-affected ROI DVR	*R*^2^ = 0.23	Mecca [110]
	**AD** + **MCI**	STT	Hip L SUVR	*r* = −0.348	Zhang [82]
	**LBD**	MoCA (visuospatial domain)	OC SUVR	*R*^2^ = 0.137	Andersen [98]
			mOC SUVR	*R*^2^ = 0.155	
**CBD symptoms**	**PSP, CBS**	CBD Rating Scale	Global BP_ND_	*r* = −0.72	Holland [86]
**PSP symptoms**	**PSP, CBS**	PSP Rating Scale	Global BP_ND_	*r* = −0.61	Holland [86]
	**PSP, CBS, bvFTD**		Striatal ICA BP_ND_ loading	*β* = −0.5	Whiteside [90]
	**PSP, CBS**	Rate of change in PSP rating scale	Δglobal BP_ND_	*r* = 0.47	Holland [91]
**Anosognosia**	**AD**	AQ-D	Hip V_T_	*r* = −0.75	Bastin [78]
			PCC V_T_	*r* = −0.43	
		MARS	Hip V_T_	*r* = −0.75	
			PCC V_T_	*r* = −0.48	
			PHG V_T_	*r* = −0.48	
			Thal V_T_	*r* = −0.48	
			pFC V_T_	*r* = −0.47	
	**bvFTD**	AQ-D	CN R V_T_	*r* = −0.89	Salmon [88]
			FP R V_T_	*r* = −0.87	
**Anxiety symptoms**	**NWPsy + OWPsy**	PSWQ	dlPFC V_T_	*r* = −0.463	Asch [67]
**Internet gaming**	**IGD**	IGD89-SF	Putamen L SUVR	*r* = −0.48	Hou [70]
			Putamen R SUVR	*r* = −0.48	
		Daily gaming	Putamen L SUVR	*r* = −0.57	
		Daily gaming	Putamen R SUVR	*r* = −0.59	
**Cocaine use**	**CoUD**	abstinence	ACC V_T_ /fp	*i* = −0.66	Angarita [68]
			VS V_T_ /fp	*r* = −0.56	
			dmPFC V_T_ /fp	*r* = −0.58	
			vmPFC V_T_ /fp	*r* = −0.61	
		frequency	ACC V_T_ /fp	*r* = 0.59	
			OFC V_T_ /fp	*r* = 0.56	
			dmPFC V_T_ /fp	*r* = 0.57	
			vmPFC V_T_ /fp	*r* = 0.58	
**Depressive symptoms**	**MDD/PTSD**	HAMD-17	ACC V_T_	*r* = −0.63	Holmes [64]
			Hip V_T_	*r* = −0.49	
			dlPFC V_T_	*r* = −0.63	
**Parkinson’s symptoms**	**PD**	MDS-UPDRS total	BS V_T_	*r* = −0.63	Wilson [95]
		MDS-UPDRS-III	BS V_T_	*r* = −0.66	
			CBL SUVR	*β* = −1.64	Van Cauwenberge [99]
			CN SUVR	*β* = −1.06	
			OC SUVR	*β* = −0.86	
			PC SUVR	*β* = −0.91	
			SN SUVR	*β* = −3.08	
			TC SUVR	*β* = −0.82	
			Thal SUVR	*β* = −2.30	
**Huntington’s symptoms**	**HD**	UHDRS motor score	Putamen SUVR	*R*^2^ = 0.67	Delva[101]
**Psychosis symptoms**	**SZ**	PANSS positive	FC BP_ND_	*r* = −0.57	Radhakrishnan [58]
		PANSS total	Hip V_T_	*r* = −0.48	Onwordi [56]
		PANSS positive	mFG R BP_ND_	*r* = −0.668	Yoon [57]

If both PVC and non-PVC results were available, then only PVC were reported in the table. Nonsignificant findings are not reported, but full data can be accessed here GitHub.

*ACC* anterior cingulate cortex, *ACE-R* Addenbrooke’s Cognitive Examination Revised, *AD* Alzheimer’s dementia, *AQ-D* Anosognosia Questionnaire – Dementia, *AVF* animal verbal fluency, *BACS* Brief Assessment of Cognition in Schizophrenia, *BNT* Boston Naming Task, *BPAD* bipolar affective disorder, *BPnd* nondisplaceable binding potential, *BS* brainstem, *(bv)FTD* (behavioural variant) frontotemporal dementia, *CaUD* cannabis use disorder, *CBD* corticobasal degeneration, *CBL* cerebellum, *CDR-SB* Clinical Dementia Scale Sum of Boxes, *CN* caudate nucleus, *CoUD* cocaine use disorder, *DMS* delayed match to sample, *DSS* digit/symbol substitution, *DVR* distribution volume ratio, *EC* entorhinal cortex, *FG* frontal gyrus, *FL* frontal lobe, *fp* plasma free fraction, *FP* frontal pole, *GAD* generalised anxiety disorder, *GM* grey matter, *HAMD-17* Hamilton Rating Scale for Depression, *HC* healthy control, *HG* Heschl’s gyrus, *Hip* hippocampus, *i* inferior, *IC* insular cortex, *ICA* independent component analysis, *IFS* INECO Frontal Screening, *IGD* internet gaming disorder, *IGDS9-SF* Nine-Item Internet Gaming Disorder Scale, *ISL* International Shopping List, *l* left, *m* medial/middle, *LBD* Lewy body dementia, *MARS* Memory Awareness Rating Scale, *MCCB* MATRICS Consensus Cognitive Battery, *MCI* mild cognitive impairment, *MDD* major depressive disorder, *MDS-UPDRS* Movement Disorder Society Unified Parkinson’s Disease Rating Scale, *MMSE* mini mental state examination, *MoCA* Montreal Cognitive Assessment, *NWPsy* normal weight with psychiatric diagnosis (MDD, PTSD, BPAD, GAD), *OC* occipital cortex, *OFC* orbitofrontal cortex, *OTC* occipitotemporal cortex, *OWPsy* overweight with psychiatric diagnosis (MDD, PTSD, BPAD, GAD), *PANSS* positive and negative symptom scale, *PC* parietal cortex, *PCC* posterior cingulate cortex, *PD* Parkinson’s disease, *(pm)HD* (premanifest) Huntington’s disease, *(dl/dm/vm)PFC* (dorsolateral/dorsomedial/ventromedial) prefrontal cortex, *PHG* parahippocampal gyrus, *PL* parietal lobe, *PSP* progressive supranuclear palsy, *PSWQ* Penn State Worry Questionnaire, *PTSD* posttraumatic stress disorder, *Put* putamen, *r* right, *RAVLT(DR)* Rey Audioverbal Learning Test (Delayed Recall), *RO* Rolandic operculum, *ROCF* Rey-Osterrieth Complex Figure, *ROI* region of interest, *s* superior, *SCWT* Stroop colour/word test, *SDMT* Symbol Digit Modalities Test, *SN* substantia nigra, *SSRT* stop signal reaction time, *STT* Shape Trail Test, *SUVR* standardised uptake value ratio, *SZ* schizophrenia, *Thal* thalamus, *TMT* Trailmaking Test, *UHDRS* Unified Huntington’s Disease Rating Scale, *(vm/dlp)FC* (ventromedial/dorsolateral pre)frontal cortex, *VS* ventral striatum, *Vt* volume of distribution, *WAIS* Weschler Adult Intelligence Scale.

Significant correlations are reported between SV2A PET measures for whole GM and several cognitive domains, including language, executive functioning, processing speed, verbal and visuospatial memory, and global cognition (Table 7). However, studies have also investigated relationship between SV2A measures in specific regions and cognitive measures; including the PFC, where lower tracer uptake was associated with deficits in executive and social functioning, processing speed, and measures of global cognitive function, and the temporal cortex, where reduced tracer uptake was associated with deficits in audioverbal memory, episodic memory, and measures of global cognition (Table 7). Whilst this might be expected for global cognitive measures, it raises the question of whether it is global or regional SV2A levels that matters most for specific aspects of cognitive performance. Table 7 shows that correlations between audioverbal learning scores and temporal and hippocampal SV2A tracer measures were stronger (*r* = 0.42–0.7) in absolute terms than correlations with frontal, insular, and ACC uptake (*r* = 0.26–0.48; Table 7), consistent with evidence that temporal regions are most strongly implicated in audioverbal learning processes [131]. Similarly, significant correlations involving executive and inhibitory functions were largely limited to the frontal lobe and ACC (Table 7). Thus, the evidence to date indicates that regional SV2A is most strongly related to region-specific aspects of cognitive performance.

### Limitations of SV2A PET

While the findings we have reviewed support the use of SV2A PET as an in vivo synaptic terminal marker, there are some issues that remain outstanding (Box 2). Additionally, we were unable to identify any research thus far to investigate the correlation between SV2A PET measures and measures of whole synapses (i.e., including postsynaptic components), so it remains unclear how closely SV2A measures are related to synaptic density, as opposed to presynaptic density. Another consideration is that altered binding of SV2A PET tracers may not be solely due to altered synaptic terminal density: alterations in SV2A copy number [132], or conformational changes in the protein itself affecting binding to tracer could affect the PET signal [133]. These issues could be addressed in future animal studies by combining in vivo PET imaging with ex vivo electron microscopy to count synapses and/or immunolabelling of pre- and postsynaptic protein markers in brain tissue. Human studies may also be feasible, particularly in neurodegenerative disorders where it may be possible to collect brain tissue postmortem. Additionally, the development of PET or other in vivo imaging measures of postsynaptic and glial markers that can be collected alongside SV2A PET data would provide a more complete picture of the synapse.

One issue relates to the reference region used in SV2A PET. The CSO has been widely used as a reference region in SV2A PET studies, however this comes with several caveats. Firstly, it is a white matter (WM) region, perfusion of which is less than half that of GM [134], leading to slower tracer delivery to WM relative to GM regions and a different time activity curve. Secondly, despite lacking synapses, the CSO does exhibit some specific binding of SV2A tracers [135], potentially leading to underestimation of GM DVR and BP_ND_. This is observed in blocking studies with levetiracetam, which find post-blockade reductions in [^11^C]UCB-J, [^18^F]SynVesT-1 and [^18^F]SynVesT-2 uptake in the CSO of 10–15%, 33.3% and 20% respectively [29, 35, 47], which would not be expected in a region lacking SV2A. It is unknown whether specific binding in the CSO may change with disease. Secondly, one study has estimated nondisplaceable uptake of [^11^C]UCB-J in the CSO at around 20% higher than GM [135], due to differing lipid contents, which may explain the paradoxical observation that using the cerebellum, which is synaptically dense [136], as a reference region gives comparable group differences to results using the CSO [74, 77]. Use of the CSO may thus be further complicated in conditions associated with WM alterations, seen in depression [137], schizophrenia [138], bipolar disorder [139], neurodegenerative illness [140], and normal ageing [141]. Indeed, Holmes et al. noted lower CSO V_T_ in MDD versus controls [64]. The CSO should therefore be seen as a pseudoreference region [142], rather than a true reference region.

Studies in this review estimated either SUVR, BP_ND_, DVR or V_T_, advantages and limitations of which are described in Box 1. Most SV2A studies using BP_ND_ estimated this via the SRTM [57, 85–87, 89–91, 104], which rests on assumptions which are violated in SV2A PET (see Schizophrenia section), and which could negatively bias results and potentially overestimate group differences [59]. Many studies used SUVR. While SUVR values at 60–90 min correlate well with DVR measures [55, 143], these studies have been performed in healthy volunteers. In one blocking study, while SUVR showed good correlation with DVR-1 at baseline, it showed a negative bias of ~20% following levetiracetam [35] compared to DVR-1, cautioning that disease effects may affect SUVR differentially. SUVR is highly sensitive to inter-individual and -group variation in tracer uptake and delivery, as it does not utilise kinetic modelling. With DVR and V_T_, the kinetic model allows estimation of tracer binding to the target at equilibrium. With SUVR, however, scans are taken within a predetermined time window after injection, meaning that variation in tracer kinetics cannot be accounted for. These factors introduce additional noise into the method, reducing power to detect changes. Indeed, use of quantitative outcome measures which allow for more sophisticated modelling has been shown to increase sensitivity [65]. In three cohorts which showed diverging results between studies, namely healthy aging, PD and MDD, this divergence was seen between studies using SUVR and those using V_T_ or other quantitative approaches as an outcome measure, with the SUVR studies reporting either nonsignificant or the more modest group differences than seen with the fully quantitative measures.

The limitations of different outcome measures should be considered when critically appraising studies, and future work to understand the expected impact of these issues on measured data and design solutions would be valuable. We do not recommend using SUVR as an outcome measure, as it is the outcome measure most subject to bias. Rather, BPND with the SRTM should be chosen if ABS is unavailable, ideally with explicit modelling of the CSO as a pseudoreference region [144]. DVR and BP_ND_ both require a suitable reference region, which has not been established for SV2A PET. We therefore recommend using VT with ABS where possible, although it should be recognised that it is subject to more noise than DVR and BP_ND_.

Finally, SV2A’s specificity for the synapse itself is not conclusive. Stockburger et al. [145] identified SV2A protein in isolated mitochondria in mice, including from outside the synapse, and found that SV2A knockdown altered mitochondrial morphology. These findings were replicated by Reichert [146], who estimates that 14.1% of SV2A colocalises with mitochondria throughout the cell, rather than being located in vesicles. They also found that mitochondrial stress, induced by the mitochondrial complex I inhibitor rotenone, promotes SV2A localisation in the mitochondrion, suggesting that [^11^C]UCB-J PET studies in diseases also associated with mitochondrial dysfunction may show greater tracer uptake in mitochondria, which could potentially lead to underestimation of loss of SV2A from the synaptic terminal. Mitochondrial SV2A may also explain the specific binding in the CSO: WM does contain mitochondria [147].

Box 2 Outstanding issues for SV2A PET validation

1. Validation studies of PET measures of synaptic protein alongside in vitro quantification are scarce. Further studies are needed to determine relationships between PET measures of SV2A and other synaptic markers measured in vitro.
2. SV2A is a presynaptic marker. Further research investigating how changes in SV2A correlate with changes in the postsynaptic density and synaptic glia are need to give a more complete picture of synaptic alterations in neuropsychiatric disorders.
3. Blocking studies mostly use levetiracetam as the displacing ligand, which may underestimate the tracers’ selectivity for the protein. Future blocking studies could use more selective ligands such as brivaracetam to further test selectivity.

### Future research directions

SV2A is expressed in all synapses so far investigated, including GABAergic [148], glutamatergic [148], and dopaminergic [149] synapses. As such, SV2A PET imaging alone cannot determine whether alterations in a given disorder are in specific synapses or generalised. Multimodal imaging studies could help elucidate the specific synaptic changes seen in neuropsychiatric illness. For example, SV2A imaging combined with MRS, or PET studies of receptor density could provide information on whether synapses in certain neurotransmitter systems are differentially affected by a disease process. Other members of the SV2 family could be potential targets for the development of novel PET tracers, as expression of these proteins may be limited to subsets of GABAergic or glutamatergic synapses [150].

Evidence presented suggests that cognitive function shows relationships with SV2A measures across disorders, and there is limited evidence of a similar trend for ratings of depression and anxiety. It would be useful to investigate the contribution of social isolation, and other potential confounds, to SV2A findings. Combining datasets to investigate correlations of symptoms across disorders with SV2A imaging measures may be useful to investigate the role of synaptic mechanisms in the expression of symptoms across neuropsychiatric illness [151] and the contribution of social factors to imaging biomarkers and symptoms. In view of this, we recommend that future SV2A PET studies report data on social, cognitive and mood measures, even if not related to the core research question.

## Conclusions

Four SV2A PET tracers with good reliability and high selectivity have been developed for human imaging to date. Studies using these tracers provide evidence that lower brain SV2A levels are associated with a number of neurological and psychiatric disorders relative to healthy controls, and with poorer cognitive function. The most replicated findings are in neurodegenerative illness, in particular tauopathies, where there is widespread lower tracer uptake relative to controls with large effect sizes in frontal cortex and hippocampus, as well as replicated findings in other regions. Amongst different psychiatric conditions studied to date, the hippocampus and PFC appear to be consistently implicated. Replicated findings in the ACC and frontal cortex are observed in chronic schizophrenia, but there are divergent findings earlier in the illness. Conflicting results are observed in major depression, although there are only two cross-sectional studies in patients of different age groups. More data is needed in both cohorts to determine whether there may be a different underlying neurobiology in later-life depression. Notwithstanding this, results from two studies suggest that drugs with antidepressant actions may increase SV2A levels. With the exception of Parkinson’s disease, lower SV2A marker levels are generally associated with greater symptom severity, in particular cognitive dysfunction. It is possible that SV2A PET may be able to uncover synaptic mechanisms for certain symptoms across different diagnoses. Overall, findings are consistent with a crucial role for synapses in a broad range of neuropsychiatric conditions, and studies in aggregate point towards several promising directions, in terms of how synaptic imaging can help understand specific illnesses, but also the overlap between them.

## Supplementary information

Supplementary information
