# Supplementary material for: Synaptic changes in psychiatric and neurological disorders: state-of-the art of in vivo imaging
Source: Neuropsychopharmacology. 2024 Aug 12;50(1):164–83. doi: 10.1038/s41386-024-01943-x (PMC11525650; doi:10.1038/s41386-024-01943-x)
Supplement: Supplementary file 1 — Supplementary information [file 41386_2024_1943_MOESM1_ESM.docx]

# Supplement

# Overview of Positron Emission Tomography

## Introduction to PET

Positron emission tomography is a molecular imaging technique that uses a bioactive tracer, labelled with a radioactive isotope, which is sequestered in a biological tissue or compartment of interest. Commonly used radioisotopes include ^11^C, ^18^F and ^15^O, choice of which depends on the chemical synthesis of the PET tracer^1^. The nucleus of the isotope is unstable, and will decay probabilistically according to its half-life, releasing a positron and a neutrino in the process. The positron travels a short distance until it encounters an electron; these annihilate each other, releasing two anti-parallel gamma photons which are detected by the PET scanner^2^. Repeat coincidental detection of these two photons, over several hundred thousand decay events, allows determination of the uptake of radiotracer in specific brain regions. Application of PET can thus measure a diverse range of physiological functions; a tracer can act as, among other things, a ligand, informing on densities of proteins^3^, receptors^4^, and neurotransmitter release^5^, as a factor in a synthetic pathway, informing on neurotransmitter synthesis^6^, or as a glucose analogue, informing on a tissue’s energy consumption^7^.

Once a target is identified, selection and validation of radiotracers is a crucial step for high-quality PET imaging. Pharmacokinetic studies will guide choice; the ideal tracer is one with rapid kinetics (i.e., a short time to maximum concentration in the brain, or T_max_), high brain uptake of the tracer with low penetrance of potentially radioactive tracer metabolites, and strong and selective binding to the biological target, which will increase sensitivity and specificity respectively^8^.

## Kinetic Modelling and Signal Quantification

A PET scanner measures the concentration of radioactivity in a voxel. The output from a PET study is a set of time-activity curves (TACs), describing how concentration of radioactivity detected by the scanner changes in a voxel as a function of time. Analysis involves fitting the TACs to a kinetic model to inform estimation of an outcome measure, which describes the uptake of tracer into the tissue of interest. The kinetic model describes the dynamic behaviour of tracer after injection. It comprises a set of ordinary differential equations defining the movement of tracer between one or more different spatial compartments in which tracer is assumed to be sequestered, e.g. blood, tissue, or in the case of receptor studies, the free and bound states^9^. The model is then fit to describe the observed PET data, and can then be solved to derive rate constants, or values describing the flow of tracer between different compartments in the model, used to calculate the study endpoints.

The kinetic model can be used with an input function, which models the delivery of radiotracer to the brain^10^. The gold-standard is direct measurement of the plasma TAC via arterial blood sampling (ABS)^11^, usually from the radial artery. This allows between-subject comparison, as the input function accounts for subject-specific factors occurring outside the brain, for example, variations in plasma clearance. However, in the absence of ABS, a reference tissue model can be used to exclude the contribution of these confounding factors from the measured signal in the region of interest (ROI)^12^. An ideal reference region exhibits minimal specific binding of the tracer to the target of interest (e.g. a receptor), but assumed-identical nondisplaceable uptake. This describes activity in the free and nonspecifically-bound compartments, such as tracer bound to macromolecular cellular components. This activity would not be reduced by adding large amounts of nonradioactive ligand into the tissue, but is assumed to relate directly to tracer delivery to brain tissue, and consequentially, the input function^13^. In the case of synaptic imaging, the centrum semiovale (CSO) is used, being a white matter region expected to lack synapses^14^, but this reference region is imperfect (see Limitations Section). Use of a reference region naturally avoids the subject burden of repeated ABS, but imposes some limitations on what endpoints can be derived from the kinetic model.

## Outcome Measures from PET Studies

The endpoints may vary with the question being investigated and its associated assumptions, and practical concerns, e.g. the availability of ABS. A simple outcome is the standardised uptake value (SUV), which is the ratio between tissue radioactivity concentration and the administered dose per unit body weight. SUV is calculated from a static PET image; i.e., it uses the average radioactivity concentration in the regions of interest over a period of time rather than dynamic changes over time. To reduce subject-specific effects, this is often presented as the SUV ratio (SUVR) between the ROI and a reference region. SUVR is a *non-quantitative* outcome measure, meaning that it does not give a value for the quantity of target, rather describes the uptake of tracer into a brain region, and does not make any assumptions about the arterial input function, making it more sensitive to intersubject variation in tracer delivery and clearance. Dynamic PET imaging allows more sophisticated modelling of the kinetics of the tracer after injection, and estimation of outcome measures less sensitive to subject-specific noise. If an arterial input function are available, volume of distribution (V_T_) can be estimated, which is the ratio of tracer concentration between the tissue and the blood. This becomes constant at equilibrium if a tracer binds reversibly to its target^3^. However, the tissue signal will include tracer bound to the target of interest, but also nondisplaceable uptake, and therefore may overestimate the measured density of the target. As such, the distribution volume ratio (DVR) is often used instead^15,16^. This is the ratio of V_T_ between the ROI and a reference region, whose signal ideally only reflects nondisplaceable uptake. The reference region approach can also be used to calculate endpoints without the need for ABS, with estimation of tracer binding potential (BP_ND_)^17^. BP_ND_ reflects both the density of available tracer binding sites and the affinity of the tracer for the protein target, and is calculated using the ratio of specific to nondisplaceable volume of distribution^18^. It can be derived from estimates of V_T_, in which case ABS will be needed, but can also be estimated using the simplified reference tissue model, whereby the arterial input function is assumed from the time activity curve of the reference region^19^.

| **Glossary of PET terminology** | |
| --- | --- |
| **Compartment** | A conceptual division where measured radioactivity could emanate from. Used to inform the compartmental model, which assumes the presence of tracer in one or more tissue compartments (e.g. blood, the free and the bound states in a receptor study). |
| **Specific uptake** | Uptake of tracer into the compartment of interest. |
| **Non-displaceable uptake** | Uptake of tracer into the free and nonspecifically bound compartments, which combines with specific uptake to constitute the measured PET signal in tissue. |
| **Reference region** | A brain region assumed to have minimal expression of the target of interest, but identical nondisplaceable uptake to other brain regions. Used to calculate various outcome measures. |
| **Standardised uptake value (SUV)** | The ratio between radioactivity concentration in a voxel and the injected dose per unit body weight. Can be used to calculate SUVR, or the ratio of SUV between a ROI and reference region, a common outcome measure, based on the average radioactivity concentration over a given period of time. |
| **Plasma free fraction (*f_p_*)** | The proportion of ligand in blood plasma not bound to plasma proteins. |
| **Volume of distribution (V_T_)** | A PET outcome measure requiring dynamic imaging, relying on measurement of time activity curves in a voxel. Describes the volume of blood that contains the same activity as 1ml of tissue in the region of interest. Dependent on measurement of the arterial input function. Sometimes given as a ratio over *f_p_* if *f_p_* varies between subjects, as this will affect the calculation of V_T_. |
| **Distribution volume ratio (DVR)** | A PET outcome measure requiring dynamic imaging, relying on measurement of time activity curves in a voxel. Describes the ratio of V_T_ between an ROI and a reference region to correct for nondisplaceable uptake. |
| **Binding potential (BP_ND_)** | A PET outcome measure requiring dynamic imaging, comprising both the affinity of tracer for the receptor and the amount of available receptor. Quantified by the ratio of activity between an ROI and reference region. |

***Table S1*** *Summary of common terminology in SV2A PET. PET = positron emission tomography. ROI = region of interest.*

| **Diagnosis** | **Reference** | **Design** | **Overlapping samples** | **Participants** | **Age mean ±SD** | **Medication** | **Radioligand** | **PET measurement** | **PET reference** |
| --- | --- | --- | --- | --- | --- | --- | --- | --- | --- |
| **HC** | **Andersen (2022)^17^** | cross-sectional | N | young=11,  old= 15 | young=24.8 ±1.2,  old= 72.7 ±4.2 | medication free | [11C]UCB-J | SUVR | CS |
|  | **Johansen (2023)^18^** | cross-sectional | subset of Langley (2023)^19^ | HC (placebo)=15, HC (escitalopram) =17 | placebo=22.8 ±2.9, escitalopram= 25.2 ±5.8 | SSRI-naive | [11C]UCB-J | VT |  |
|  | **Michiels (2021)^20^** | cross-sectional | HC: Delva (2020)^21^ | HC=78 | HC (median)=55 | medication free | [11C]UCB-J | SUVR | CS |
|  | **Toyonaga (2023)^22^** | cross-sectional | HC: Chen (2018)^23^, Finnema (2018), Holmes (2019)^24^, Finnema (2020)^25^, Matuskey (2020)^26^, Mecca (2020)^27^, D'Souza (2019)^28^ | HC=80 | HC (range)= 21–83 | SSRI-naive | [11C]UCB-J | SUVR, BPf | CS |
|  | **Van Aalst (2021)^29^** | cross-sectional | N | HC=20 | HC= 29.6 ± 9.9 | medication free | [11C]UCB-J | BPND | CS |

**Table S2** Summary of studies in healthy controls. If the same sample or a subset of the same sample was used across multiple studies, this is indicated in the ‘Overlapping samples’ column, where citation indicates the first study published (chronologically) where this sample was included; otherwise ‘N’ indicates no overlap in data with previous studies. **Abbreviations**: CS = centrum semiovale, HC = healthy control, N = no, PET = positron emission tomography, SD = standard deviation, SSRI = selective serotonin reuptake inhibitor, SUVR = standardised uptake value ratio, Vt = volume of distribution.

| **Diagnosis** | **Reference** | **Design** | **Overlapping samples** | **Participants** | **Age mean ±SD** | **Medication** | **Radioligand** | **PET measurement** | **PET reference** |
| --- | --- | --- | --- | --- | --- | --- | --- | --- | --- |
| **Addictions** | **Angarita (2022)^30^** | cross-sectional | 8 HC: Finnema (2018)^31^, Holmes (2019)^34^, Finnema (2019)^31^, Rossano (2020)^32^ | HC=15,  CoUD=15 | HC=43.4 ± 9.2, CoUD= 42.9 ± 7.5 | not reported | [^11^C]UCB-J | VT/fp, VT, BPND | CS |
|  | **D’Souza (2019)** ^28^ | cross-sectional | N | HC=12,  CaUD=12 | HC=27.32 ±4.41, CaUD 24.24 ±3.12 | not reported | [11C]UCB-J | VT, BPND | CS |
|  | **Hou (2023)**^33^ | cross-sectional | N | HC=16,  IGD=18 | HC=22.44 ±5.44, IGD=20.53 ±3.69 | all medication-naive | [18F]SynVesT-1 | SUVR | CS |
| **Mood disorders** | **Asch (2022)**^34^ | cross-sectional | N | HC: normal weight (NWHC, n=16), overweight (OWHC, n=28), normal weight (NWPsy, n=14), ovewrweight patients (OWPsy, n=16) | NWHC=41.94 ±4.00,  NWPsy= 40.77 ±2.68,  OWHC= 46.50 ±5.03,  OWCPsy= 37.46 ±2.27 | NWPsych (n=7), OWPsych (n=8) | [11C]UCB-J | VT, VT/fp | n/a |
|  | **Casteele (2023)**^35^ | cross-sectional | NK | HC=36, Depression=24 | HC=70.4 ± 6.2, Depression=73.0 ± 6.2 | not reported | [11C]UCB-J | SUVR | CS |
|  | **Holmes (2019)**^24^ | cross-sectional | N | HC=21, MDD/PTSD=26 (14 low + 12 high severity) | HC=44.48 ±15.41, low= 39.14 ±11.18, high=38.67 ±13.89 | medication free (4m) | [11C]UCB-J | VT | n/a |
|  | **Holmes (2022)**^36^ | cross-sectional | N | HC=9, MDD/PTSD=12 (6 SV2A deficit, 6 no SV2A deficit) | HC=35.11 ±12.68, MDD/PTSD= 39.91 ±11.23 | in clinical group: Paxil (n=1), Wellbutrin (n=2) | [11C]UCB-J | VT | n/a |
| **SZ** | **Onwordi (2020)**^37^ | cross-sectional | N | HC=18,  SZ=18 | HC=38.7±3.1,  SZ=41.5 ±2.7 | most taking antipsychotics | [11C]UCB-J | DVR, VT | CS |
|  | **Onwordi (2021)**^38^ | cross-sectional | Onwordi (2020)^37^ | HC=22,  SZ=18 | HC=38.23 ±2.59, SZ=40.89 ±2.75 | not reported | [11C]UCB-J | VT, DVR | CS |
|  | **Onwordi (2023)**^39^ | cross-sectional | Onwordi (2020)^37^ | HC=21,  HC=21 | HC=30.86 ±1.90, SZ=26.52 ±1.74 | Antipsychotic naïve (n=2), antipsychotic-free (n=19) | [11C]UCB-J | DVR, VT, VT/fp | CS |
|  | **Radhakrishnan (2021)**^40^ | cross-sectional | N | HC=15,  SZ=13 | HC=40.77 ±11.04, SZ=40.52 ±11.15 | antipsychotic-free (n=3) | [11C]UCB-J | VT, BPND | CS |
|  | **Yoon (2023)**^41^ | cross-sectional | N | HC=9,  SZ=9 | HC=27.22 ±4.60, SZ=25.67 ±3.97 | taking antipsychotics (n=8), antipsychotic-free (n=1) | [11C]UCB-J | BPND | CS |

**Table S3** Summary of studies in psychiatric illnesses. If the same sample or a subset of the same sample was used across multiple studies, this is indicated in the ‘Overlapping samples’ column, where citation indicates the first study published (chronologically) where this sample was included; otherwise ‘N’ indicates no overlap in data with previous studies. **Abbreviations**: BP_ND_ = nondisplaceable binding potential, CaUD = cannabis use disorder, CoUD = cocaine use disorder, CS = centrum semiovale, DVR = distribution volume ratio, fp = plasma free fraction, HC = healthy controls, IGD = internet gaming disorder, MDD = major depressive disorder, N = no, NA = not applicable, NK = not known, NWHC = normal weight healthy control, NWPsy = normal weight with psychiatric diagnosis, OWHC = overweight healthy control, OWPsy = overweight with psychiatric diagnosis, PTSD = posttraumatic stress disorder, SUVR = standardised uptake value ratio, SZ = schizophrenia, V_T_ = volume of distribution.

| **Diagnosis** | **Reference** | **Design** | **Overlapping samples** | **Participants** | **Age mean ±SD** | **Radioligand** | **PET measurement** | **PET reference** |
| --- | --- | --- | --- | --- | --- | --- | --- | --- |
| **AD** | **Bastin (2020)**^42^ | cross-sectional | N | Aβ+ patients=25, HC=21 | Aβ+=73.3 ±8.0, HC=71.5 ±4.5 | [18F]-UCB-H | Vt | CS |
|  | **Chen (2018)**^23^ | cross-sectional | N | AD=10 (MCI=5, mild AD=5), HC=11 | AD=72.7 ±6.3,, HC=72.9 ±8.7 | [11C]-UCB-J | VT, BPND | CS |
|  | **Chen (2021)**^43^ | cross-sectional | Chen (2018)^23^ | AD=14 (MCI=10, AD=4), HC=11 | AD=69 ±5.7,  HC=69.4 ±9.0 | [11C]-UCB-J,  [18F]-FDG | DVR | CS |
|  | **Coomans (2021)**^44^ | cross-sectional | N | AD=7 | AD=64.3 ±8.2 | [11C]-UCB-J,  [18F]-flortaucipir | BPND | CS |
|  | **Lu (2021)**^45^ | cross-sectional | Chen (2018)^23^ | AD=17 (dementia=6, MCI=11), HC=11 | between 58-82 | [11C]-PiB,  [11C]-UCB-J | Vt, BPND | CS |
|  | **Mecca (2020)**^27^ | cross-sectional | Chen (2018)^23^ | Aβ+=34 (dementia=20, MCI=14), HC=19 | Aβ+=71.7 ±8.2, HC=70.2 ±7.9 | [11C]-PiB, [11C]-UCB-J | BPND, DVRcs | CS, CBL |
|  | **Mecca (2022a)**^46^ | cross-sectional | Chen (2018)^23^ | AD=10 (dementia=5, MCI=5), HC=10 | AD=72.1 ±7.9,  HC=68.8 ±6.6 | [11C]-UCB-J,  [18F]-flortaucipir | DVR | CS, CBL |
|  | **Mecca (2022b)^47^** | cross-sectional | Chen (2018)^23^ | AD=45 (dementia=28, MCI=17),HC=19 | AD=70.82 ±7.48, HC=70.84 ±7.78 | [11C]-UCB-J | DVR | CS |
|  | **Moallemian (2023)**^48^ | cross-sectional | Bastin (2020)^42^ | AD=24, HC=19 | AD=73.08 ±8.07, HC=71.95 ±4.58 | [18F]-UCB-H | Vt | ? |
|  | **O’Dell (2021)**^49^ | cross-sectional | Chen (2018)^23^ | MCI=14, AD=24, HC=19 | MCI=71.6 ±4.2, AD=69.9 ±9.2,  HC=71.5 ±7.6 | [11C]-UCB-J,  [11C]-PiB | DVR | CBL |
|  | **O’Dell (2023)**^50^ | cross-sectional | Chen (2018)^23^ | HC=18, AD=45 (MCI=17, dementia=28) | HC=70.8 ±7.8,  AD=70.8 ±7.5 | [11C]-UCB-J,  [11C]-PiB | DVR | CBL |
|  | **Tuncel (2021)^20^** | test-retest | N | AD=8, HC=9 | AD=63.8, HC=62.2 | [11C]UCB-J | BP_ND_ | CS |
|  | **Silva-Rudberg (2024)**^51^ | cross-sectional | Chen (2018)^23^ | AD MCI=14, AD-mild dementia=19, HC=17 | AD=70.3 ±7.7, HC=72.1 ±7.1 | [11C]-UCB-J,  [11C]-PiB | DVR | CBL |
|  | **Venkataraman (2022)**^52^ | cross-sectional | N, pre-print: Venkataraman (2021)^53^ | AD=12 (8 followed-up), HC=16 | AD=75.50 ±7.82, HC=65.88 ±9.82 | [11C]-UCB-J | DVRcs | CS |
|  | **Zhang (2023)**^54^ | cross-sectional | N | AD=33, MCI=31, HC=30 | AD=68.0 ±7.8, MCI=70.1 ±6.8, HC=63.4 ±8.0 | [18]F-Syn-VesT-1 | SUVR | CBL |
| **MCI** | **Vanderlinden (2022)**^55^ | cross-sectional, longitudinal | follow up to Vanhaute (2020)^56^ | aMCI=26, HC=12 | aMCI=71.6 ±5.3, HC=68.9 ±9.1 | [11C]-UCB-J, [11C]SA4503, [18F]BCPP-EF | SUVR | CS |
|  | **Vanhaute (2020)**^56^ | cross-sectional | N | aMCI=10, HC=10 | aMCI=69.6 ±5.4, HC=67.2 ±6.5 | [11C]-UCB-J,  [18]F-MK-6241, [11C]-PiB | SUVR | CS,  inferior CBL Cx |
| **bvFTD** | **Malpetti (2022)**^57^ | cross-sectional | N | bvFTD=11, HC=25 | bvFTD=65.7 ±9.3, HC=70.2 ±7 | [11C]-UCB-J | BPnd | CS |
| **FTD/AD** | **Salmon (2021)**^58^ | cross-sectional | Bastin (2020)^42^ | bvFTD=12, HC=12, AD=12 | bvFTD=73.5,  AD=74.1, HC=71.4 | [18F]-UCB-H | Vt | NA, used image-derived AIF |
| **HD** | **Delva (2022a)**^59^ | cross-sectional, longitudinal | N | HD=18, (pmHD=7, mHD=11), HC=15 | HD=51.4 ±11.6 (pmHD=45.0 ±11.6, mHD=55.2 ±9.6), HC=52.3 ±3.5 | [11C]-UCB-J, [18F]-FDG | SUVR | CS |
|  | **Delva (2023)**^60^ | cross-sectional, longitudinal | Delva (2022a)^59^ | HD=17 (pmHD=6, mHD=11), HC=13 | (at followup) HC=54.4 ±3.7,  HD=52.9 ±12.2 | [11C]-UCB-J, [18F]-FDG | SUVR-1 | CS |
| **LBD** | **Nicastro (2020)**^61^ | cross-sectional | N | LBD=2 (Aβ+: 1, Aβ-:1), HC=10 | LBD=73,  HC 72.4 ±3.4 | [11C]-UCB-J,  [18F]-AV1451,  [11C]-PiB | BPND | CBL GM |
| **LBD, PD** | **Andersen (2021)**^62^ | cross-sectional | N | HC=15, nPD=21, DLB/PDD=13(9/4) | HC=72.4 ±4.2, nPD=71.1 ±6.4, DLB/PDD=74.3 ±4.7 | [11C]-UCB-J | SUVR-1 | CBL WM, CS |
|  | **Andersen (2023)**^63^ | cross-sectional | Andersen (2021)^62^ | FDG PET included: HC=15, nPD=29, DLB/PDD=7(3/4) | HC=72.4 ±4.2, nPD=71.1 ±6.4, DLB/PDD=74.3 ±4.7 | [11C]-UCB-J, [18F]-FDG | SUVR-1 | CS |
| **PD** | **Delva (2020)**^21^ | cross-sectional | N | PD=30, HC=20 | PD=60.5 ±9.2,  HC=59.6 ±8.2 | [11C]-UCB-J, [18F]-FE-PE21 | BPND | CS |
|  | **Delva (2022b)**^64^ | cross-sectional, longitudinal | Delva (2020)^21^ | PD=27, HC=18 | PD=62.7 ±9.8,  HC=61.3 ±8.6 | [18F]-FE-PE21, [11C]-UCB-J | SUVR-1 | CS, Ocx |
|  | **Matuskey (2020)**^26^ | cross-sectional | N | PD=15, HC=15 | PD=76(7),  HC=84(7) | [11C]-UCB-J | BPnd | CS |
|  | **Wilson (2020)**^65^ | cross-sectional, longitudinal | N | PD=12, HC=16 | PD=59.8 ±9,  HC=61 ±12.5 | [11C]-UCB-J,  [18F]-BCCP-EF,  [11C]-SA-4503 | Vt | CS |
| **PSP, CBS, bvFTD** | **Whiteside (2023)**^66^ | cross-sectional | Holland (2020)^71^ | PSP=29, CBS=16, bvFTD=10, HC=24 | PSP=70.8±8.4, CBS=67.1±5.7, bvFTD=65±9.1, HC=70±8.4 | [11C]-UCB-J | BPnd | CS |
| **Tauopathies (PSP, amyloid-negatve CBS)** | **Holland (2020)**^71^ | cross-sectional | N | PSP=14, CBS=9, HC=15 | PSP=72.8 ±7.7, CBD=70.6 ±8.2,  HC=60 ±7.45 | [11C]-UCB-J | BPND | CS |
|  | **Holland (2022)**^67^ | cross-sectional | Holland (2020)^71^ | PSP=23, CBS=12, HC=19 | PSP=71.3 ±8.6, CBD=70.9±7.9, HC=68.9±7.1 | [11C]-UCB-J,  [18F]-AV-1451 | BPND | CS, Inferior CBL grey matter |
|  | **Holland (2023)**^68^ | cross-sectional, longitudinal | Holland (2020)^71^ | PSP=32, CBD=16, HC=33, Followup PSP=16, Followup CBD=6, Followup HC=0 | PSP=71.7±8.24, CBD=70.7±7.2, HC=71±8.5, Followup PSP=72.1±8.1, Followup CBD=71±10.8 | [11C]-UCB-J | BPnd | CS |
|  | **Mak (2021)**^69^ | cross-sectional | N | PSP=22, CBS=14, HC=27 | PSP=70.9 ±8.69, CBS=70 ±7.91,  HC=69 ±7.34 | [11C]-UCB-J | BPnd | CS |
| **TLE** | **Finnema (2020)**^25^ | cross-sectional | N | TLE=12, HC=12 | TLE=39.4 ±10.7,  HD=40 ±12 | [11C]-UCB-J,  [18F]-FDG | BPND | CS |
| **MMS** | **Van Cauwenberge (2023)**^70^ | cross-sectional | Delva (2020)^21^ | HC=58 | HC=68.5 ±7.5 | [11C]-UCB-J | SUVR | CS |

**Table S4** Summary of studies in neurodegenerative illnesses. If the same sample or a subset of the same sample was used across multiple studies, this is indicated in the ‘Overlapping samples’ column, where citation indicates the first study published (chronologically) where this sample was included; otherwise ‘N’ indicates no overlap in data with previous studies. **Abbreviations**: Aβ = beta-amyloid, AD = Alzheimer’s dementia, AIF = arterial input function, BP_ND_ = nondisplaceable binding potential, (bv)FTD = (behavioural variant) frontotemporal dementia, CBD = corticobasal degeneration, CBL = cerebellum, CS = centrum semiovale, Cx = cortex, DVR = distribution volume ratio, GM = grey matter, HC = healthy controls, LBD = Lewy body dementia, MCI = mild cognitive impairment, MMS = mild motor signs, N = no, NA = not applicable, OCx = occipital cortex, PD = Parkinson’s disease, (pm)HD = (premanifest) Huntington’s disease, PSP = progressive supranuclear palsy, SUVR = standardised uptake value ratio, TLE = temporal lobe epilepsy, V_T_ = volume of distribution, WM = white matter.

# References

1. Miller PW, Long NJ, Vilar R, Gee AD. Synthesis of 11C, 18F, 15O, and 13N radiolabels for positron emission tomography. *Angewandte Chemie International Edition*. 2008;47(47):8998-9033.

2. Turkington TG. Introduction to PET instrumentation. *Journal of nuclear medicine technology*. 2001;29(1):4-11.

3. McCluskey SP, Plisson C, Rabiner EA, Howes O. Advances in CNS PET: the state-of-the-art for new imaging targets for pathophysiology and drug development. *European journal of nuclear medicine and molecular imaging*. 2020;47:451-489.

4. Rogeau A, Nordio G, Veronese M, et al. The relationship between glutamate, dopamine, and cortical gray matter: a simultaneous PET-MR study. *Molecular Psychiatry*. 2022;27(8):3493-3500.

5. Abi-Dargham A, Kegeles LS, Zea-Ponce Y, et al. Striatal amphetamine-induced dopamine release in patients with schizotypal personality disorder studied with single photon emission computed tomography and [123I] iodobenzamide. *Biological psychiatry*. 2004;55(10):1001-1006.

6. Howes OD, Bose SK, Turkheimer F, et al. Dopamine synthesis capacity before onset of psychosis: a prospective [18F]-DOPA PET imaging study. *American Journal of Psychiatry*. 2011;168(12):1311-1317.

7. Schöll M, Damián A, Engler H. Fluorodeoxyglucose PET in neurology and psychiatry. *PET clinics*. 2014;9(4):371-390.

8. Laruelle M, Slifstein M, Huang Y. Relationships between radiotracer properties and image quality in molecular imaging of the brain with positron emission tomography. *Molecular Imaging & Biology*. 2003;5(6):363-375.

9. Morris EE, Christopher J; Schmidt, Kathleen C; Christian, Bradley T; Muzic, Raymond F; Fisher, Ronald E. Kinetic Modeling in Positron Emission Tomography. *Emission Tomography*. Elsevier; 2004:499-540:chap 23.

10. Bentourkia Mh. Determination of the input function at the entry of the tissue of interest and its impact on PET kinetic modeling parameters. *Molecular Imaging and Biology*. 2015;17:748-756.

11. Wong K-P, Feng D, Meikle SR, Fulham MJ. Simultaneous estimation of physiological parameters and the input function-in vivo PET data. *Ieee transactions on information technology in biomedicine*. 2001;5(1):67-76.

12. Gunn RN, Lammertsma AA, Hume SP, Cunningham VJ. Parametric imaging of ligand-receptor binding in PET using a simplified reference region model. *Neuroimage*. 1997;6(4):279-287.

13. Zanderigo F, Ogden RT, Parsey RV. Reference region approaches in PET: a comparative study on multiple radioligands. *Journal of Cerebral Blood Flow & Metabolism*. 2013;33(6):888-897.

14. Rossano S, Toyonaga T, Finnema SJ, et al. Assessment of a white matter reference region for 11C-UCB-J PET quantification. *Journal of Cerebral Blood Flow & Metabolism*. 2020;40(9):1890-1901.

15. Mecca AP, Chen MK, O'Dell RS, et al. In vivo measurement of widespread synaptic loss in Alzheimer's disease with SV2A PET. *Alzheimer's & Dementia*. 2020;16(7):974-982.

16. Silva-Rudberg JA, Salardini E, O'Dell RS, et al. Assessment of Gray Matter Microstructure and Synaptic Density in Alzheimer's Disease: A Multimodal Imaging Study With DTI and SV2A PET. *The American Journal of Geriatric Psychiatry*. 2023;

17. Mintun MA, Raichle ME, Kilbourn MR, Wooten GF, Welch MJ. A quantitative model for the in vivo assessment of drug binding sites with positron emission tomography. *Annals of Neurology: Official Journal of the American Neurological Association and the Child Neurology Society*. 1984;15(3):217-227.

18. Innis RB, Cunningham VJ, Delforge J, et al. Consensus nomenclature for in vivo imaging of reversibly binding radioligands. *Journal of Cerebral Blood Flow & Metabolism*. 2007;27(9):1533-1539.

19. Lammertsma AA, Hume SP. Simplified reference tissue model for PET receptor studies. *Neuroimage*. 1996;4(3):153-158.

20. Tuncel H, Boellaard R, Coomans EM, et al. Kinetics and 28-day test–retest repeatability and reproducibility of [11C] UCB-J PET brain imaging. *Journal of Cerebral Blood Flow & Metabolism*. 2021;41(6):1338-1350.

17. Andersen, K. B. *et al.* Healthy brain aging assessed with [18F]FDG and [11C]UCB-J PET. *Nucl. Med. Biol.* **112–113**, 52–58 (2022).

18. Johansen, A. *et al.* Effects of escitalopram on synaptic density in the healthy human brain: a randomized controlled trial. *Mol. Psychiatry* (2023) doi:10.1038/s41380-023-02285-8.

19. Langley, C. *et al.* Chronic escitalopram in healthy volunteers has specific effects on reinforcement sensitivity: a double-blind, placebo-controlled semi-randomised study. *Neuropsychopharmacology* **48**, 664–670 (2023).

20. Michiels, L. *et al.* Synaptic density in healthy human aging is not influenced by age or sex: a 11C-UCB-J PET study. *NeuroImage* **232**, 117877 (2021).

21. Delva, A., Van Weehaeghe, D., Koole, M., Van Laere, K. & Vandenberghe, W. Loss of Presynaptic Terminal Integrity in the Substantia Nigra in Early Parkinson’s Disease. *Mov. Disord.* **35**, 1977–1986 (2020).

22. Toyonaga, T. *et al.* The regional pattern of age-related synaptic loss in the human brain differs from gray matter volume loss: in vivo PET measurement with [11C]UCB-J. *Eur. J. Nucl. Med. Mol. Imaging* (2023) doi:10.1007/s00259-023-06487-8.

23. Chen, M.-K. *et al.* Assessing Synaptic Density in Alzheimer Disease With Synaptic Vesicle Glycoprotein 2A Positron Emission Tomographic Imaging. *JAMA Neurol.* **75**, 1215 (2018).

24. Holmes, S. E. *et al.* Lower synaptic density is associated with depression severity and network alterations. *Nat. Commun.* **10**, 1529 (2019).

25. Finnema, S. J. *et al.* Reduced synaptic vesicle protein 2A binding in temporal lobe epilepsy: A [ 11C]UCB‐J positron emission tomography study. *Epilepsia* **61**, 2183–2193 (2020).

26. Matuskey, D. *et al.* Synaptic Changes in Parkinson Disease Assessed with in vivo Imaging. *Ann. Neurol.* **87**, 329–338 (2020).

27. Mecca, A. P. *et al.* In vivo measurement of widespread synaptic loss in Alzheimer’s disease with SV2A PET. *Alzheimers Dement.* **16**, 974–982 (2020).

28. D’Souza, D. C. *et al.* Preliminary in vivo evidence of lower hippocampal synaptic density in cannabis use disorder. *Mol. Psychiatry* **26**, 3192–3200 (2021).

29. Van Aalst, J. *et al.* In vivo synaptic density relates to glucose metabolism at rest in healthy subjects, but is strongly modulated by regional differences. *J. Cereb. Blood Flow Metab.* **41**, 1978–1987 (2021).

30. Angarita, G. A. *et al.* Lower prefrontal cortical synaptic vesicle binding in cocaine use disorder: An exploratory 11C‐UCB‐J positron emission tomography study in humans. *Addict. Biol.* **27**, e13123 (2022).

31. Finnema, S. J. *et al.* A single‐center, open‐label positron emission tomography study to evaluate brivaracetam and levetiracetam synaptic vesicle glycoprotein 2A binding in healthy volunteers. *Epilepsia* **60**, 958–967 (2019).

32. Rossano, S. *et al.* Assessment of a white matter reference region for ^11^ C-UCB-J PET quantification. *J. Cereb. Blood Flow Metab.* **40**, 1890–1901 (2020).

33. Hou, J. *et al.* Lower synaptic density associated with gaming disorder: an ^18^ F-SynVesT-1 PET imaging study. *Gen. Psychiatry* **36**, e101112 (2023).

34. Asch, R. H. *et al.* Lower synaptic density is associated with psychiatric and cognitive alterations in obesity. *Neuropsychopharmacology* **47**, 543–552 (2022).

35. Casteele, T. V. *et al.* 69. Lower Grey Matter Volume is not Related to Synaptic Density in Late Life Depression. *Biol. Psychiatry* **93**, S121–S122 (2023).

36. Holmes, S. E. *et al.* Imaging the effect of ketamine on synaptic density (SV2A) in the living brain. *Mol. Psychiatry* **27**, 2273–2281 (2022).

37. Onwordi, E. C. *et al.* Synaptic density marker SV2A is reduced in schizophrenia patients and unaffected by antipsychotics in rats. *Nat. Commun.* **11**, 246 (2020).

38. Onwordi, E. C. *et al.* The relationship between synaptic density marker SV2A, glutamate and N-acetyl aspartate levels in healthy volunteers and schizophrenia: a multimodal PET and magnetic resonance spectroscopy brain imaging study. *Transl. Psychiatry* **11**, 393 (2021).

39. Onwordi, E. C. *et al.* Synaptic Terminal Density Early in the Course of Schizophrenia: An In Vivo UCB-J Positron Emission Tomographic Imaging Study of Synaptic Vesicle Glycoprotein 2A. *Biol. Psychiatry* S0006322323013537 (2023) doi:10.1016/j.biopsych.2023.05.022.

40. Radhakrishnan, R. *et al.* In vivo evidence of lower synaptic vesicle density in schizophrenia. *Mol. Psychiatry* **26**, 7690–7698 (2021).

41. Yoon, J. H. *et al.* Reductions in synaptic marker SV2A in early-course Schizophrenia. *J. Psychiatr. Res.* **161**, 213–217 (2023).

42. Bastin, C. *et al.* In vivo imaging of synaptic loss in Alzheimer’s disease with [18F]UCB-H positron emission tomography. *Eur. J. Nucl. Med. Mol. Imaging* **47**, 390–402 (2020).

43. Chen, M.-K. *et al.* Comparison of [11C]UCB-J and [18F]FDG PET in Alzheimer’s disease: A tracer kinetic modeling study. *J. Cereb. Blood Flow Metab.* **41**, 2395–2409 (2021).

44. Coomans, E. M. *et al.* In vivo tau pathology is associated with synaptic loss and altered synaptic function. *Alzheimers Res. Ther.* **13**, 35 (2021).

45. Lu, Y. *et al.* Partial volume correction analysis for 11C-UCB-J PET studies of Alzheimer’s disease. *NeuroImage* **238**, 118248 (2021).

46. Mecca, A. P. *et al.* Association of entorhinal cortical tau deposition and hippocampal synaptic density in older individuals with normal cognition and early Alzheimer’s disease. *Neurobiol. Aging* **111**, 44–53 (2022).

47. Mecca, A. P. *et al.* Synaptic density and cognitive performance in Alzheimer’s disease: A PET imaging study with [11C]UCB‐J. *Alzheimers Dement.* **18**, 2527–2536 (2022).

48. Moallemian, S. *et al.* Multimodal imaging of microstructural cerebral alterations and loss of synaptic density in Alzheimer’s disease. *Neurobiol. Aging* **132**, 24–35 (2023).

49. O’Dell, R. S. *et al.* Association of Aβ deposition and regional synaptic density in early Alzheimer’s disease: a PET imaging study with [11C]UCB-J. *Alzheimers Res. Ther.* **13**, 11 (2021).

50. O’Dell, R. S. *et al.* Principal component analysis of synaptic density measured with [11C]UCB-J PET in early Alzheimer’s disease. *NeuroImage Clin.* **39**, 103457 (2023).

51. Silva-Rudberg, J. A. *et al.* Assessment of Gray Matter Microstructure and Synaptic Density in Alzheimer’s Disease: A Multimodal Imaging Study With DTI and SV2A PET. *Am. J. Geriatr. Psychiatry* **32**, 17–28 (2024).

52. Venkataraman, A. V. *et al.* Widespread cell stress and mitochondrial dysfunction occur in patients with early Alzheimer’s disease. *Sci. Transl. Med.* **14**, eabk1051 (2022).

53. Venkataraman, A. V. *et al.* *Imaging Synaptic Microstructure and Synaptic Loss* in Vivo *in Early Alzheimer’s Disease*. http://medrxiv.org/lookup/doi/10.1101/2021.11.23.21266746 (2021) doi:10.1101/2021.11.23.21266746.

54. Zhang, J. *et al.* In vivo synaptic density loss correlates with impaired functional and related structural connectivity in Alzheimer’s disease. *J. Cereb. Blood Flow Metab.* **43**, 977–988 (2023).

55. Vanderlinden, G. *et al.* Spatial decrease of synaptic density in amnestic mild cognitive impairment follows the tau build-up pattern. *Mol. Psychiatry* **27**, 4244–4251 (2022).

56. Vanhaute, H. *et al.* In vivo synaptic density loss is related to tau deposition in amnestic mild cognitive impairment. *Neurology* **95**, (2020).

57. Malpetti, M. *et al.* Synaptic Loss in Frontotemporal Dementia Revealed by [11C]UCB‐J Positron Emission Tomography. *Ann. Neurol.* **93**, 142–154 (2022).

58. Salmon, E. *et al.* In vivo exploration of synaptic projections in frontotemporal dementia. *Sci. Rep.* **11**, 16092 (2021).

59. Delva, A., Michiels, L., Koole, M., Van Laere, K. & Vandenberghe, W. Synaptic Damage and Its Clinical Correlates in People With Early Huntington Disease: A PET Study. *Neurology* **98**, (2022).

60. Delva, A., Van Laere, K. & Vandenberghe, W. Longitudinal Imaging of Regional Brain Volumes, SV2A, and Glucose Metabolism In Huntington’s Disease. *Mov. Disord.* **38**, 1515–1526 (2023).

61. Nicastro, N. *et al.* 11C-UCB-J synaptic PET and multimodal imaging in dementia with Lewy bodies. *Eur. J. Hybrid Imaging* **4**, 25 (2020).

62. Andersen, K. B. *et al.* Reduced Synaptic Density in Patients with Lewy Body Dementia: An [11C]UCB‐J PET Imaging Study. *Mov. Disord.* **36**, 2057–2065 (2021).

63. Andersen, K. B. *et al.* Synaptic Density and Glucose Consumption in Patients with Lewy Body Diseases: An [11C]UCB-J and [18F]FDG PET Study. *Mov. Disord.* **38**, 796–805 (2023).

64. Delva, A., Van Laere, K. & Vandenberghe, W. Longitudinal Positron Emission Tomography Imaging of Presynaptic Terminals in Early Parkinson’s Disease. *Mov. Disord.* **37**, 1883–1892 (2022).

65. Wilson, H. *et al.* Mitochondrial Complex 1, Sigma 1, and Synaptic Vesicle 2A in Early Drug-Naive Parkinson’s Disease. *Mov. Disord.* **35**, 1416–1427 (2020).

66. Whiteside, D. J. *et al.* Synaptic density affects clinical severity via network dysfunction in syndromes associated with frontotemporal lobar degeneration. *Nat. Commun.* **14**, 8458 (2023).

67. Holland, N. *et al.* Molecular pathology and synaptic loss in primary tauopathies: an 18F-AV-1451 and 11C-UCB-J PET study. *Brain* **145**, 340–348 (2022).

68. Holland, N. *et al.* Longitudinal Synaptic Loss in Primary Tauopathies: An In Vivo [ ^11^ C ] UCB‐J Positron Emission Tomography Study. *Mov. Disord.* **38**, 1316–1326 (2023).

69. Mak, E. *et al.* In vivo coupling of dendritic complexity with presynaptic density in primary tauopathies. *Neurobiol. Aging* **101**, 187–198 (2021).

70. Van Cauwenberge, M. G. A. *et al.* Mild Motor Signs in Healthy Aging Are Associated with Lower Synaptic Density in the Brain. *Mov. Disord.* **38**, 1786–1794 (2023).

71. Holland, N. P. *et al.* Synaptic loss in primary tauopathies revealed by [11C] UCB-J positron emission tomography. *Mov. Disord.* **35**, 1834-1842 (2020).
